# Tracking the Evolution of Single-Atom Catalysts for the CO_2_ Electrocatalytic Reduction Using Operando X-ray Absorption Spectroscopy and Machine Learning

**DOI:** 10.1021/jacs.3c04826

**Published:** 2023-07-31

**Authors:** Andrea Martini, Dorottya Hursán, Janis Timoshenko, Martina Rüscher, Felix Haase, Clara Rettenmaier, Eduardo Ortega, Ane Etxebarria, Beatriz Roldan Cuenya

**Affiliations:** Department of Interface Science, Fritz-Haber Institute of the Max Planck Society, 14195 Berlin, Germany

## Abstract

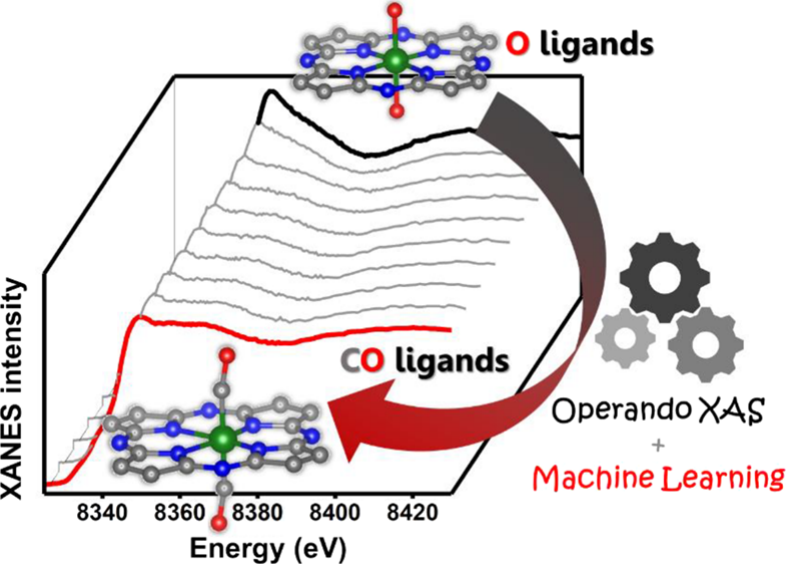

Transition metal-nitrogen-doped carbons (TMNCs) are a promising class of catalysts for the CO_2_ electrochemical reduction reaction. In particular, high CO_2_-to-CO conversion activities and selectivities were demonstrated for Ni-based TMNCs. Nonetheless, open questions remain about the nature, stability, and evolution of the Ni active sites during the reaction. In this work, we address this issue by combining operando X-ray absorption spectroscopy with advanced data analysis. In particular, we show that the combination of unsupervised and supervised machine learning approaches is able to decipher the X-ray absorption near edge structure (XANES) of the TMNCs, disentangling the contributions of different metal sites coexisting in the working TMNC catalyst. Moreover, quantitative structural information about the local environment of active species, including their interaction with adsorbates, has been obtained, shedding light on the complex dynamic mechanism of the CO_2_ electroreduction.

## 1 Introduction

The CO_2_ electrocatalytic reduction reaction (CO_2_RR), powered by renewable electricity sources, is a promising pathway for the abatement of the CO_2_ emissions from industrial sites, where concentrated CO_2_ is available, and for the associated production of valuable chemical feedstocks. Nonetheless, suitable active and selective catalysts are still needed.^1^ Transition metal nitrogen-doped carbons (TMNCs) have attracted attention as promising electrocatalysts for CO_2_RR due to their high activity and selectivity for the CO_2_ conversion to CO, especially for Ni-based TMNCs.^2−4^ The structure and the catalytic functionality of these systems differ significantly from those of bulk or nanostructured materials.^5−7^ In TMNCs, nitrogen atoms are incorporated in the carbon matrix forming binding sites for the metal species. These singly dispersed metal sites are often considered to be the active species for the CO_2_RR reaction,^8−11^ as well as for other electrochemical processes such as the oxygen reduction reaction (ORR).^7,12−15^ Nonetheless, in addition to singly dispersed metal sites, a large number of different structural motifs can coexist during the reaction in TMNC catalysts, hindering the unambiguous identification of the active species.^16^ These can include multiple pyrollic and pyridinic nitrogen environments, metallic clusters and carbide, and oxide and nitride particles.^2^ We especially highlight here that the species present under the reaction conditions and participating in the catalytic processes can differ significantly from those in the as-prepared samples, as showcased by the striking example of Cu-based TMNCs, for which the reversible formation of metallic Cu clusters under CO_2_RR conditions was recently reported.^17,18^ Thus, only an operando characterization can provide unambiguous answers about the active state of the TMNC catalysts.^5,19−21^ However, considering the low metal loadings, the lack of long-range ordering in TMNC materials, as well as their heterogeneous nature, only a few experimental techniques are up to this task.^6,22^

X-ray absorption spectroscopy (XAS) stands out as an element-specific tool that can be applied to a broad range of functional materials under working conditions.^5,20,21^ Thanks to its element-selectivity, XAS can be employed for the determination of the oxidation state and local atomic and electronic structure of the metal sites, shedding light on the possible active moieties for the CO_2_RR. In particular, the analysis of the extended X-ray absorption fine structure (EXAFS) was shown to be an invaluable tool for the confirmation of the singly dispersed nature of the TMNC catalysts and for the identification of M–N_*x*_ moieties (where the *x* denotes the average number of N atoms bound to the metal site M; usually *x* is equal to 4) as the main building block for the TMNC catalysts, at least in the as-prepared state.^23−28^

On the other hand, in the absence of clusters and more ordered structural motifs, the information content in the EXAFS spectra is limited. In this situation, the X-ray absorption near edge structure (XANES) part of the XAS spectrum can provide the decisive evidence about the nature of the active states. In comparison to EXAFS, XANES is more sensitive to the symmetry of the metal center, to the 3D geometry of its environment and distortions.^5^ Moreover, XANES spectra have typically a higher signal-to-noise ratio as compared to EXAFS data, making them more suitable for time-resolved operando investigations, when the data quality in the EXAFS region is often compromised by the low metal loading and signal attenuation by the electrolyte or the elements composing the electrochemical cell. Further adoption of XANES analysis for quantitative investigations of working catalysts, however, is hindered by the lack of simple fitting approaches. Nonetheless, during the last years, with the development of reliable XANES ab initio simulation approaches, machine learning methods, and DFT modeling,^7,13,21,29−36^ the situation has begun to change, making quantitative XANES-based analysis for TMNC materials finally feasible. Despite this initial progress, most of the existing studies have focused on understanding the structure of the as-prepared TMNC catalysts. Moreover, the few existing operando XANES studies were devoted mostly to the ORR reaction.^7,13,37^ Little attention has been paid so far to the fact that the actual working TMNC catalysts could feature coexisting metal sites with different nonequivalent environments, rendering the commonly used fitting approaches, which assume a single structural model, inaccurate.

Here, operando time-resolved XANES data were used to unveil the local structure around Ni sites not simply in their as-prepared or “after reaction” states but under realistic working conditions during CO_2_RR. A multistep approach has been used here. First, we identified the number of different coexisting Ni species, their corresponding kinetic profiles, and XANES spectra using unsupervised machine learning methodologies, such as the principal component analysis (PCA) combined with a transformation matrix technique.^21,38^ In a second step, we deduced the atomistic structures for each of the identified species through a XANES fitting procedure realized by exploiting a supervised machine learning approach.^21,30^ Finally, we validate the predicted structures processing the corresponding EXAFS spectra via Reverse Monte Carlo (RMC) simulations,^39−41^ taking into account the structural disorder in the local environment around the identified Ni species.

## 2 Experimental Section

### 2.1 Sample Preparation

Ni-based TMNC catalysts were synthesized following an impregnation-calcination method as reported in the literature.^42^ First, we prepared a zeolitic-imidazolate framework (ZIF-8) precursor in the reaction between zinc-nitrate (Zn(NO_3_)_2_·6H_2_O, 98%, Acros Organics) and 2-methylimidazole (C_4_H_6_N_2_, 99% Sigma-Aldrich). Specifically, we dissolved 6.78 g of Zn(NO_3_)_2_·6 H_2_O and 7.87 g of 2-methylimidazole in 800 mL of methanol (CH_3_OH, ≥99.8%, Honeywell), heated the solution to ca. 60 °C, and stirred for 24 h under reflux. Next, we collected the ZIF-8 crystals by centrifugation and thoroughly washed them two times with methanol and once with ethanol. The obtained crystals were dried in air at 60 °C and subjected to carbonization in Ar flow at 1000 °C for 1 h. At this temperature, most of the metallic Zn evaporates from the sample, leaving behind a porous (Zn)-N-doped carbon structure (denoted as N–C). To remove all the crystalline (not single-atomic) Zn species from the N-C support, we performed an acid washing at room temperature using 20 wt% nitric-acid (HNO_3_, ≥ 65%, Carl Roth) over 24 h, followed by a thorough washing and vacuum filtration of the sample with ultrapure water (at least 3 × 600 mL) until the pH of the supernatant solution reached a value larger than 5. After drying the acid-washed N–C, we impregnated it with a solution of nickel-nitrate (Ni(NO_3_)_2_·6H_2_O, Sigma-Aldrich, 99.999%). 200 mg of N–C was added to a solution of 20 mL of 6 mM Ni(NO_3_)_2_ in isopropanol, the suspension was sonicated for 2 h in an ultrasonic bath at ∼30 to 40 °C and then stirred for another 2 h with a magnetic stirrer at room temperature. We obtained the “precursor” sample denoted here as Ni-TMNC after centrifugation. To get the final catalyst, which we refer to as HT-Ni-TMNC, the precursor was subjected to another heat treatment in Ar flow at 700 °C. Finally, the HT-Ni-TMNC was washed with ultrapure water, centrifuged, and dried in air at 60 °C. The prepared samples were analyzed ex situ using transmission electron microscopy (TEM), X-ray diffraction (XRD), and X-ray photoelectron spectroscopy (XPS).

For the electrochemical and operando XAS experiments, the catalysts were deposited onto carbon paper supports (Freudenberg H15C13, Fuel Cell Store). The catalyst ink consisted of 30 mg catalyst (Ni-TMNC or HT-Ni-TMNC), 75 μL Nafion 117 solution (5%, Sigma-Aldrich), 2.4 mL ultrapure water, and 2.4 mL isopropanol (C_3_H_7_OH, ≥99.8%, Sigma-Aldrich). After sonicating the ink for at least 60 min, it was spray-coated onto the preheated (90 °C) carbon paper until the desired mass loading was reached (1.2 ± 0.1 mg cm^–2^ for the electrocatalytic measurements, while 1.6 ± 0.1 mg cm^–2^ was used for the operando XAS measurements).

### 2.2 Ex Situ Characterization

#### 2.2.1 Transmission Electron Microscopy

TEM images were acquired using a probe-corrected JEM-ARM 200F (JEOL, Japan) scanning transmission electron microscope (STEM) equipped with a cold field emission gun (CFEG) operated at 200 kV. The high angle annular dark field (HAADF), annular bright field (ABF), and bright field (BF) detector signals were collected from an electron probe with a 14.2 mrad convergence semiangle and a 90–370, 12–40, 18 mrad collection semiangle, respectively. The beam current was kept at 11 pA and its resulting electron dose was scaled by the pixel size. Image acquisition and manipulation were performed with the DigitalMicrograph software v2.4 (Gatan, USA).

Energy-dispersive X-ray spectroscopy (EDS) spectra and elemental mapping were acquired using a Talos F200X (ThermoFisher Scientific, USA) STEM microscope operated at 200 kV and equipped with four silicon drift detectors (SDDs). The 72 pA electron beam with a 10.5 mrad probe convergence semiangle was scanned across the region of interest under a continuous frame acquisition mode. The EDS quantification was performed using the Velox software v1.4.2 (ThermoFisher Scientific). To reduce the background signal of the carbon framework, the net elemental maps (baseline intensity counts removed) were displayed to highlight the presence of the doping heavy metals.

For the postreaction TEM analysis, the CO_2_RR experiments were performed using glassy carbon plates as catalyst supports to avoid the presence of carbon originating from the carbon paper.

#### 2.2.2 X-ray Diffraction

Powder X-ray diffractograms were recorded with a Bruker D8 Advance instrument using a Cu anode (8046.3 eV) between 10 and 90° 2Θ values, with 0.02° step size and 3 s dwell time.

#### 2.2.3 X-ray Photoelectron Spectroscopy (XPS)

XPS spectra were acquired with a SPECS Phoibos 150 spectrometer with an Al Kα source (300 W, 12.52 kV). Survey spectra were recorded with 100 eV pass energy, 0.1 s dwell time, 0.75 eV step size, and 2 scans. The N 1s and the metal 2p regions were recorded with 30 eV pass energy, 0.3 s dwell time, and 0.15 eV step size using 20 and 60 scans, respectively. Data analysis and fitting were performed using the CasaXPS software. High-resolution spectra were fitted with 70% Gaussian and 30% Lorentzian line shapes, and a Shirley background subtraction was applied. The binding energy scale was adjusted by assigning the signal of graphitic carbon to 285 eV. The FWHM for N peaks was fixed between 1.5 and 1.6, and the spectra were fitted based on previously established protocols for similar materials.^43−48^

#### 2.2.4 Inductively Coupled Plasma Mass Spectrometry (ICP-MS)

Catalysts were first digested in a microwave digestion system (Anton Paar, Multiwave GO) at 180 °C for 20 min in an acid mixture containing cc. HNO_3_, cc. H_2_SO_4_, cc. HCl in a volume ratio of 2:2:6. Then, the solutions were filtered and diluted to ca. 50 mL with ultrapure water. For the ICP-MS measurement, a 20× dilution of each sample in 3% HNO_3_ was prepared. The measurements were performed with a ThermoScientific iCAP RQ instrument.

### 2.3 Electrocatalytic Activity Measurements

The electrochemical measurements were performed using an Autolab PGSTAT302N potentiostat/galvanostat. The working electrode was the catalyst-coated carbon paper, usually with a 0.5–1 cm^2^ geometric surface area. The counter electrode was a Pt-mesh and the potentials were measured against a leak-free Ag/AgCl electrode (0.242 V vs standard hydrogen electrode). We report the potentials throughout the text versus the reversible hydrogen electrode (RHE). These were calculated using the following equation: *E* (vs RHE) = *E* (vs Ag/AgCl) + 0.242 + 0.059 × pH. The solution resistance (*R*_u_) for the IR correction was determined by electrochemical impedance spectroscopy using the high-frequency intercept of the semicircle on the Nyquist plot with the real axis. The IR-corrected potential (*E*_–IR_) was calculated with the following formula *E*_–IR_ = *E* – *I*·*R*_u_, where *E* is the applied/measured potential, while *I* is the applied/measured current.

CO_2_RR experiments were performed in a gas-tight two-compartment H-type cell. Its description and schematic depiction are given in Section S1 and Figure S1. The cathode and anode compartments were separated by a Selemion anion exchange membrane to avoid product mixing. CO_2_ was continuously bubbled through the anolyte and catholyte with 20 mL min^–1^ flow rate. The electrolysis was performed in a 0.1 M KHCO_3_ solution that was pretreated with an ion-exchange resin (Chelex 100 Resin sodium form; Bio-Rad) to remove metal impurities. The gas outlet of the cathode compartment was directly connected to the injector of the gas chromatograph via a 6-port valve, allowing the online detection of the gaseous products. Samples were automatically injected every 15 min of the reaction. Gas products were detected and quantified by an Agilent 7890B gas chromatograph. The products were separated by different columns (Molecular sieve 13×, HayeSep Q, and Carboxen-1010 PLOT) and subsequently quantified with a flame ionization detector (FID) as well as with a thermal conductivity detector (TCD).

In the liquid phase, acetate and formate concentrations were analyzed by high-performance liquid chromatography (HPLC, Shimadzu prominence) equipped with a NUCLEOGEL SUGAR 810 column and refractive index detector (RID). Other liquid products (alcohols and aldehydes) were quantified with a liquid GC (L-GC, Shimadzu 2010 plus) equipped with a fused silica capillary column and FID detector.

### 2.4 Operando XAS Measurements

We acquired operando Ni K-edge XAS data at the BESSY II synchrotron (KMC-3 XPP beamline).^49^ Operando XAS measurements were performed in our home-built single-compartment electrochemical cell.^5^ The schematic depiction of the cell and its description can be found in Section S1 and Figure S2 in the Supporting Information (SI). Measurements were performed in a CO_2_-saturated 0.1 M KHCO_3_ electrolyte under a static current of −10 mA, which corresponds to a current density of −15.7 mA/cm^2^. A Pt mesh was used as a counter electrode, while a leak-free Ag/AgCl electrode constituted the potential reference. The applied current was controlled by a *BioLogic* potentiostat. We collected time-resolved spectra with an acquisition rate of ca. 9 min per spectrum until no further changes could be observed in the XANES data. Because of the ultra-dispersed nature of the catalysts, the latter parameter was the minimal total time necessary to acquire the entire XAS signal, sampling properly the XANES pre-edge and white line regions, and measuring the corresponding EXAFS part with a good signal-to-noise ratio. We emphasize that the collection of the XANES part of each XAS spectrum took just a fraction (ca. 1 min) of the total acquisition time, making XANES a more reliable probe of rapid processes associated with the changes in the catalyst structure.

We performed the alignment, the background subtraction, and the normalization of the corresponding XAS profiles using the Athena software.^50^ For the further processing of the XANES spectra, we applied supervised and unsupervised machine learning approaches provided by the PyFitIt code.^51^ Finally, we realized the EXAFS data fitting employing Reverse Monte Carlo simulations^52^ using the EvAX code,^39,41^ exploiting the structural models deriving from the XANES data analysis as the initial guesses for the EXAFS-based 3D structural refinement of the catalyst structure.

## 3 Results

### 3.1 Ex Situ Characterization and CO_2_RR Activity

**Figure 1:**
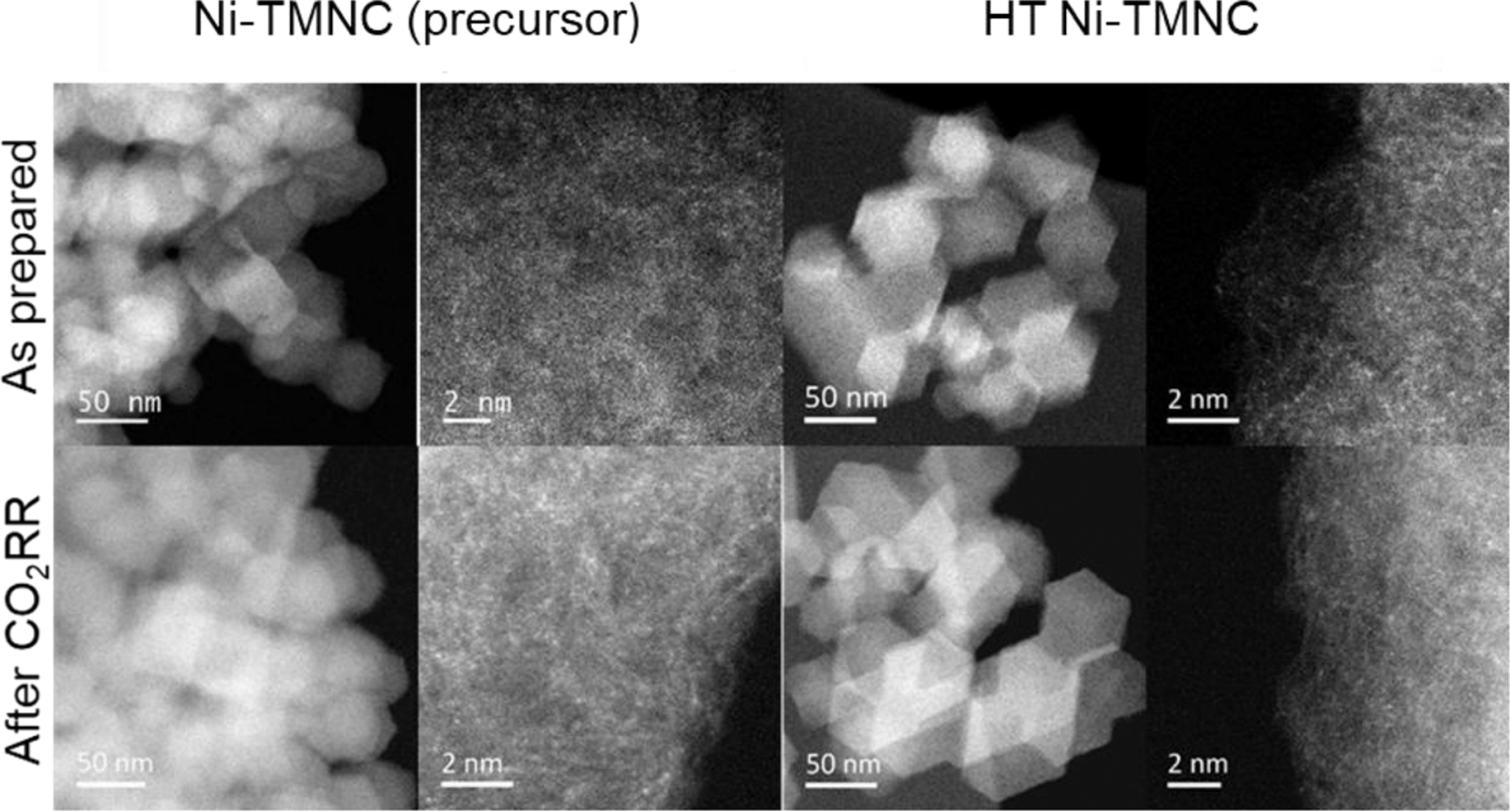
STEM annular dark field (ADF) images of the nickel-nitrogen-doped carbon catalysts. The high-resolution HR-STEM images show the disordered carbon structure and the presence of individual metal atoms (bright spots) before and after CO_2_RR.

To characterize the materials, we first collected the powder X-ray diffractograms of the precursor (Ni-TMNC) and heat-treated (HT-Ni-TMNC) catalyst. The XRD patterns for both samples are typical for an amorphous carbon (see Figure S3). The two broad reflexions at around 25° and 43° are related to the (002) and (101) planes of graphite. Importantly, no reflections indicative of the presence of crystalline phases were detected. To unambiguously prove the absence of Ni/NiO nanoparticles and Zn/ZnO nanoparticles remaining from the N-C support preparation, we carefully investigated the materials using TEM. The medium-resolution HAADF-STEM images in Figure 1 show the rhomboid-dodecahedron morphology of the N–C carbon, which was inherited from the ZIF-8 precursor. No nanoparticles were observed in these materials. In the high-resolution images, the single metal atoms appear as brighter dots, because of the higher Z-contrast of the heavy elements, compared to that of the carbon/nitrogen atoms. From these images, however, we cannot tell whether these are Ni or Zn single atoms, as Zn remaining from the ZIF-8 precursor is also inherently present in the materials. Nevertheless, energy-dispersive elemental mapping revealed the uniform distribution of Ni in our samples, without any visible agglomeration (Figure S4). This is a strong indication that Ni is present as single atoms in the as-prepared state of the catalysts, which is further confirmed by our detailed XAS analysis provided below.

The elemental composition of the catalysts was analyzed by XPS and ICP-MS (Figure S5 and Tables S1 and S2). The main components of the catalysts were C (85–90%), N (6–7%), and O (3.5–7%). The Zn and Ni contents were below 0.5 at %. Interestingly, the Ni-TMNC (precursor sample) displayed a lower Ni content and also had a lower Ni/N ratio as compared to the heat-treated catalyst. We fitted the high-resolution N-1s spectra (Figure S6a,b and Table S3) with components typically observed for MNC materials.^43−48^ The main N species were pyridinic N (398.3 eV), followed by N–H (i.e., pyrrolic or hydrogenated pyridinic N at 400.8 eV) for both catalysts. The peak at 399.5 eV is indicative of nitrogen-coordinated metal sites. We also note that Ni-TMNC contained significantly larger amounts of N–O_*x*_ groups, compared to HT-Ni-TMNC (16.9 vs 6.8%), indicating that the nitrate groups originating from the Ni(NO)_3_ decomposed or reduced during the heat treatment at 700 °C. We also recorded the high-resolution Ni 2p regions of the catalysts. Because of the low metal loading, hence weak signal intensity, we could not perform accurate peak deconvolution using the multiple fitting features of Ni oxides and hydroxides. Instead, we focused on the position of the main peaks to determine the dominant oxidation state of Ni present in the samples.^3^ The 2p_3/2_ main peak is located at either 855.2 or 855.5 eV for the HT-Ni-TMNC and the Ni-TMNC samples, respectively, which is close to the main peak of Ni(OH)_2_ reported in the literature.^53^ Thus, the oxidation state of Ni is +2 in these samples. It should be noted that Ni-phthalocyanines (NiPc) also have the main XPS line between 854.8 and 855.2 eV;^54−56^ hence, the main peak of HT Ni-TMNC may also (partially) originate from a porphyrin-like structure. In the spectrum of HT Ni-TMNC, we also observed a weak shoulder at 852 eV (2% of the main peak), originating from the negligible amounts of Ni^0^. The heat treatment of the Ni-TMNC catalyst resulted in a notable reduction in the satellite intensity (by ∼35%), which may also suggest an increasing contribution from a NiPc-like structure in the HT Ni-TMNC catalyst.^3^

**Figure 2:**
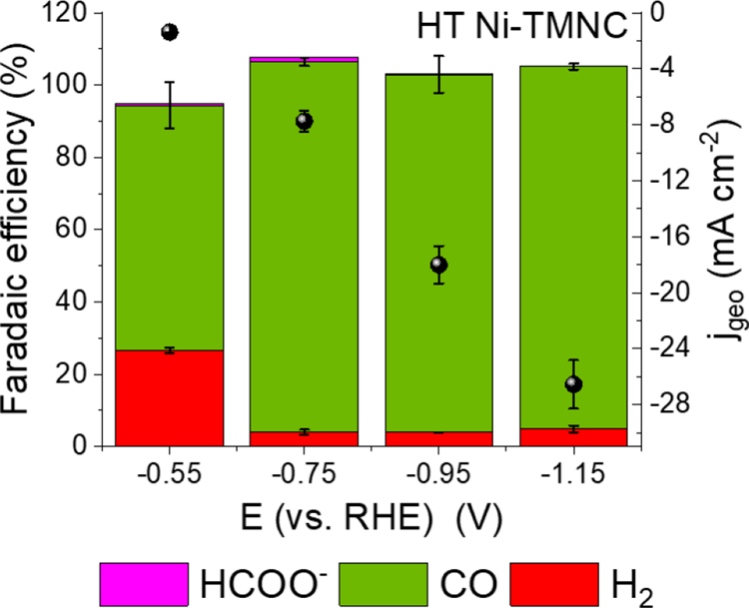
Catalytic activity (total current densities) and selectivities (faradaic efficiencies) of the HT-Ni-TMNC catalysts in the CO_2_RR. Catalytic tests were performed under potential control in an H-type cell using a CO_2_-saturated 0.1 M KHCO_3_ electrolyte. The currents were normalized by the geometric surface area. Error bars reflect the standard deviation of the measured data for measurements performed on at least two separate electrodes.

We tested the activity of the catalysts in the CO_2_RR in an H-type cell configuration in CO_2_-saturated 0.1 M KHCO_3_ electrolyte between −0.55 and −1.15 V (*vs* RHE), see Figure 2 for the HT-Ni-TMNC and Figure S7 for the Ni-TMNC. The potential-dependent catalytic activity is also provided. Both catalysts produced CO with high selectivity (>90% faradaic efficiency at potentials below −0.6 V), accompanied by only minor hydrogen evolution as a side reaction, in accordance with previous reports on Ni single atomic catalysts.^2−4^ In terms of current density, the heat-treated catalyst, however, outperformed the precursor sample. At −0.95 V, the total current density was −18 mA cm^–2^ for HT-Ni-TMNC, while only −11 mA cm^–2^ for Ni-TMNC. In addition, the CO partial current density decayed more rapidly over time in the case of Ni-TMNC (Figure S8), indicating that the heat treatment at 700 °C stabilized the Ni single sites. Importantly, no nanoparticle or agglomerate formation was observed by TEM analysis after reaction in any of the samples (see Figure 1).

### 3.2 Qualitative XANES and EXAFS Analysis

**Figure 3:**
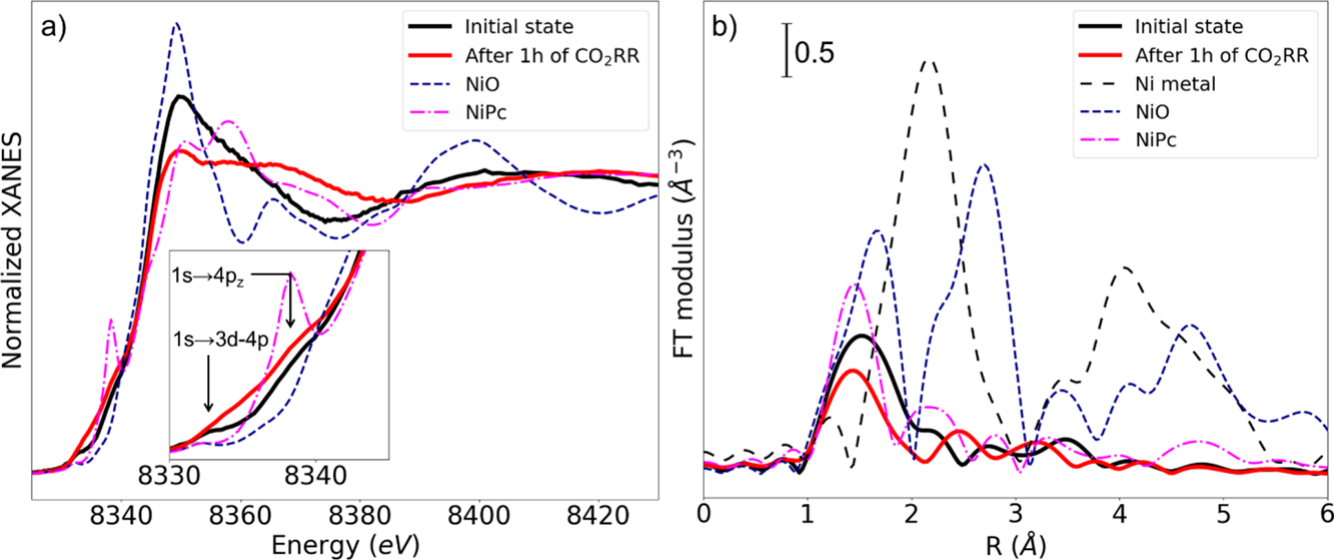
Operando Ni K-edge (a) XANES and (b) magnitudes of the Fourier Transformed (FT) EXAFS (phase uncorrected) spectra collected before and at the end of the CO_2_RR (after 1 h) for the HT Ni-TMNC sample. The inset in panel (a) shows a magnification of the XANES pre-edge region. The Fourier transform of the EXAFS spectra was carried out in the *k* space between 1 and 9 Å^–1^. XAS measurements were performed in a CO_2_-saturated 0.1 M KHCO_3_ electrolyte under a static current density of −15.7 mA/cm^2^.

For the operando XAS experiments, the CO_2_RR was performed under galvanostatic conditions with −15.7 mA cm^–2^ applied current density (Figure S9). Figure 3a shows Ni K-edge XANES spectra collected for the HT-Ni-TMNC catalyst at the beginning and at the end of the CO_2_RR process, while the complete set of XANES spectra collected under CO_2_RR conditions can be found in Figure S10. One can immediately note that the XANES spectra experience significant changes, suggesting strong transformations in the catalyst structure. In all cases, the position of the absorption edge matches well that of reference materials with Ni species in the 2+ state. At the same time, the XANES features both, for the initial catalyst and its final state, differ clearly from those in the available reference spectra for NiO and NiPc, suggesting that the local structure around the Ni sites is distinct from that in these standard materials.

The initial spectrum for the HT-Ni-TMNC catalyst is characterized by a relatively strong main XANES feature at ca. 8350 eV (the so-called white line, W.L.), which resembles the W.L. in the reference spectrum for the rocksalt-type NiO. Nonetheless, for our catalyst, the W.L. feature is broader and less intense than the W.L. of the NiO compound. Under the CO_2_RR conditions, the W.L. feature further decreases, while the two main pre-edge peaks located at ca. 8333 and 8338 eV acquire progressively more intensity. By comparing the spectra of the HT-Ni-TMNC sample with that of the NiPc reference and with the available literature data for similar systems,^57^ we can assign these pre-edge features to the 1s → 3d–4p (quadrupole) and the 1s → 4p_z_ (dipole allowed-shakedown contribution) transitions, respectively. Such pre-edge features are characteristics of planar Ni complexes. The 1s → 4p_z_ feature that gets enhanced under CO_2_RR conditions is considered to be a fingerprint of the square-planar Ni–N_4_ configuration with a *D*_4h_ symmetry.^25,58^ The intensity of this feature diminishes with the distortion of the square-planar environment. At the same time, such geometrical distortions result in an enhancement of the 1s → 3d–4p peak.^26,59−61^ Thus, based on a visual examination of the operando spectra, we can hypothesize that the changes in the Ni K-edge XANES spectra under CO_2_RR are associated with a loss of the octahedral geometry of the Ni(II) sites characteristic for the as-prepared samples, leading to the formation of distorted square planar Ni–N_4_ units, although the formation of a squared pyramidal or trigonal bi-pyramidal structures cannot be ruled out, as shown in refs (58, 62). Herein, it is also worth noting that during CO_2_RR we did not observe any significant shift of the absorption edge position (see Figure S11a), suggesting that the Ni oxidation state does not change. This evidence allows us to rule out the formation of metallic Ni clusters during the reaction, in accordance with the post reaction TEM analysis.

The qualitative findings from the XANES data are corroborated with those derived from the examination of the EXAFS data. The raw EXAFS data are reported in Figure S11b, while Figure 3b shows the magnitude of the Fourier-transformed (FT) EXAFS signals. In the as-prepared state, the FT-EXAFS spectra are dominated by the contribution of the first coordination shell (single peak at ca. 1.5 Å, phase uncorrected). The intensity and position of this peak resemble those in the reference spectrum for NiO, suggesting similar octahedral coordination. The lack of strong peaks at larger *R*-values confirms the single-site nature of the catalyst, as also highlighted by the corresponding wavelet transforms of the EXAFS spectra reported in Section S4 of the SI. Nonetheless, caution is needed when the sample-averaging EXAFS method is used to draw such conclusions since it cannot rule out the presence of small fractions of disordered clusters.^63,64^ The smaller peaks at 2.5 and 3.7 Å in the FT-EXAFS data can be tentatively attributed to the interactions between Ni and the carbon support. Under CO_2_RR conditions, the intensity of the main FT-EXAFS peak decreases, while the intensities of the second and third peak increase, supporting the hypothesis of significant rearrangements in the local environment of Ni sites that, nonetheless, still preserve their singly dispersed nature. All FT-EXAFS peaks also appear to shift to lower *R*-values, indicating a contraction of the interatomic distances between Ni species and their nearest neighbors.

### 3.3 Spectral Decomposition of the XANES Dataset

Quantitative information about the type and relative amount of distinct species appearing and evolving during a chemical reaction can often be retrieved by the analysis of the corresponding XANES spectra employing the linear combination fitting. Following this approach, every spectrum of the XANES dataset can be written as a linear combination of certain XANES standards, which must be selected beforehand.^21,38^ This approach, however, cannot be applied if the structural motifs contributing to the experimental data are not known and/or differ significantly from the structures of well-defined standard compounds.^65^ This is clearly the case in the studies of TMNC catalysts, where the unique catalytic functionality is ensured by the presence of unique structural motifs with no analogues among the common bulk standard materials. In the absence of suitable references, the decomposition of experimental XANES data in a set of spectra for pure compounds and associated concentration profiles is still possible and can be realized by means of unsupervised machine learning methods. Here, as a first step, it is necessary to identify the number of spectroscopically distinct species present during the reaction. Afterward, the XANES spectra corresponding to these pure chemical species can be deduced from the original series of experimental spectra, providing that (i) these species exhibit sufficient spectroscopic contrast and (ii) that their concentration profiles are linearly independent (i.e., the ratios of different species are different for at least several collected spectra).

In our case, we focused on the analysis of a set of XANES data (Figure 3a) consisting of nine spectra collected for the HT-Ni-TMNC sample during the CO_2_RR process. This set of discretized spectra in the energy region between *E*_min_ = 8325 and *E*_max_ = 8430 eV forms a matrix **X**. The objective of our analysis is to decompose this matrix in terms of spectra for pure species and associated concentrations, forming the data matrices **S** and **C**, respectively: **X** = **SC**. The number of distinct chemical species appearing and evolving during the chemical reaction can be determined by the principal component analysis (PCA). The dataset **X** can be expressed as **X = UΣV**. Here, the columns of matrix **U** are orthonormal (energy-dependent) vectors, sorted by their importance to the original dataset (the amount of the XANES variance accounted by the respective component). We refer to these vectors as principal components (PCs). **Σ** is a diagonal matrix whose elements, called singular values, characterize the importance of each PC in the dataset **X**. Finally, the rows of matrix **V** contain the projections of each spectrum in the dataset **X** on the corresponding PC vector (scaled by the corresponding singular value). We emphasize that the PCs themselves do not correspond to the spectra of any particular chemical species. However, all the spectra in the dataset **X**, as well as all the spectra of the pure compounds can be represented as some linear combinations of the PCs. The number of significant PCs (i.e., those with significant corresponding singular values) thus defines the dimensionality of the dataset **X** and corresponds to the number of spectroscopically distinct species present during the reaction. By analyzing the PCs and their corresponding singular values, we concluded that, in our case, three PCs are sufficient to explain all the variations in our original dataset **X**. Indeed, we saw that only the first three PCs have some distinct features that can be linked to particular features in our experimental XANES spectra. The fourth and the subsequent PCs encode the experimental noise and bear no structural information (see Figure S13 of Section S5). This evidence leads us to conclude that the number of independent Ni species present in our catalyst is three. A more detailed discussion of the determination of a number of species based on PCA is given in Section S5 and Figures S14 and S15.

**Figure 4:**
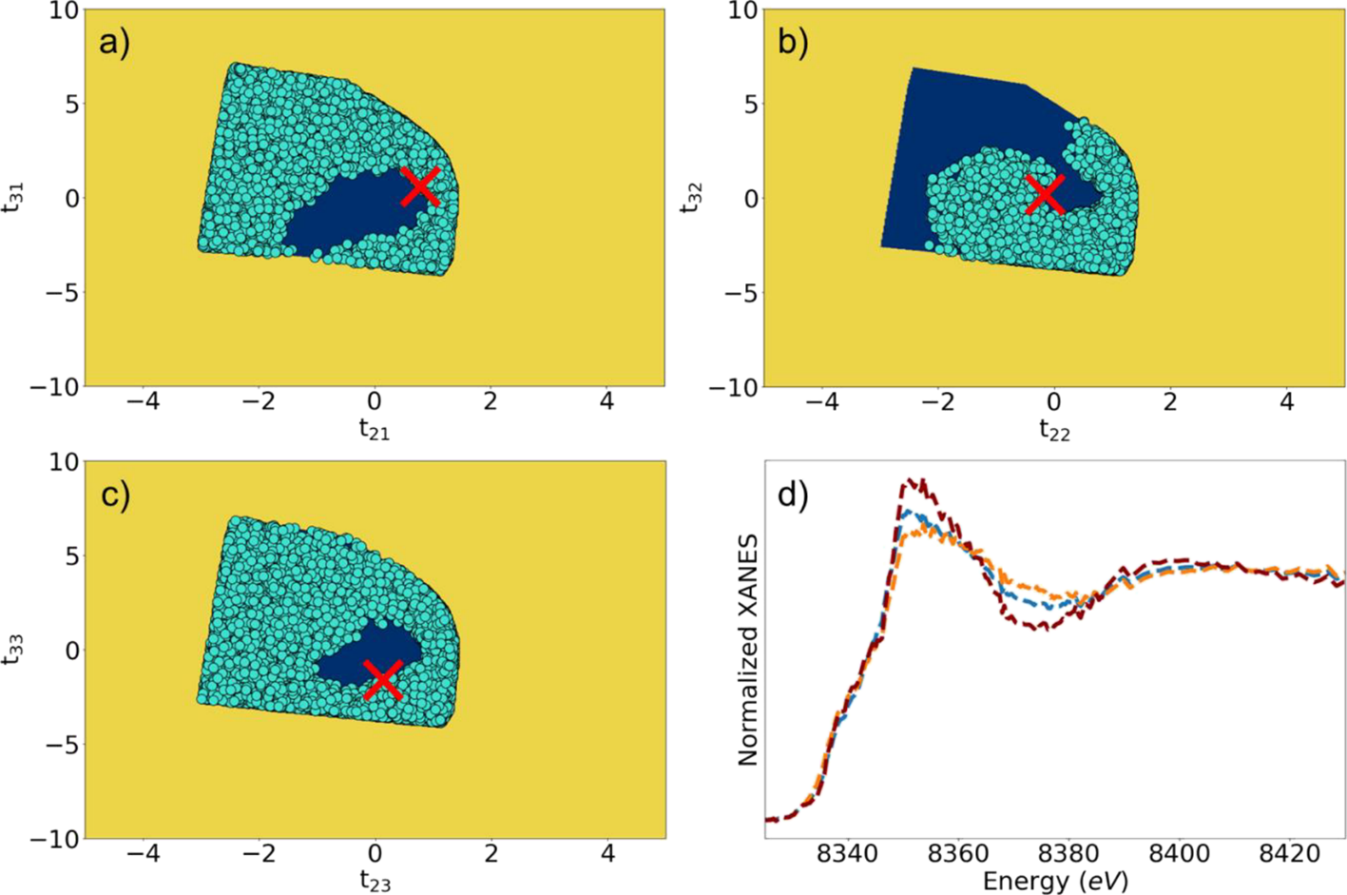
(a–c) Dark blue polygons: areas of feasible solutions obtained using a spectra-specific constraint requiring that XANES spectra are nonnegative and have a limited maximal amplitude. The light blue circles show narrower AFS obtained imposing as a further constraint that all the concentration values should be numbers between 0 and 1. Panel (a) corresponds to the first pure species, while (b, c) to the second and the third ones, respectively. The three XANES spectra in panel (d) are examples of feasible XANES spectra for the second species, corresponding to three different solutions depicted in panel (b). The red crosses indicate the single point solution, where it is assumed, in addition, that the first and the last spectrum in the experimental dataset (i.e., the spectrum corresponding to the as-prepared sample in air and the final spectrum collected under the CO_2_RR conditions) correspond to pure species.

Knowing that only three species are present in our sample during CO_2_RR, we can write X≈**ŨΣ̃Ṽ**, where we approximate the initial dataset **X** using only the first three columns of **U**, the first three singular values of **Σ** and the first three rows of **V**. As a next step, to retrieve a set of XANES spectra corresponding to the pure compounds, we follow the transformation matrix (TM) approach as implemented in PyFitIt.^66^ We note that the decomposition of matrix **X** can be further rewritten as **X** ≈ **ŨΣ̃TT^*–*1^V**∼, where **I** = **TT^*–*1^** is a 3 × 3 identity matrix. The role of matrix **T** is to transform the abstract PCs in the matrix **Ũ** into the set of actual XANES spectra corresponding to pure species: **S** = **ŨΣ̃T**. The remaining part of the decomposition, **C** = **T^*–*1^Ṽ**, will then correspond to the set of concentration profiles. The problem now is how to determine the nine unknown elements of the 3×3 matrix **T**. The number of unknowns can be decreased to six by introducing the mass balance condition (i.e., the condition that the sum of all concentrations of all species should be equal to 1) together with ensuring the proper normalization of the pure spectra components. In PyFitIt, this step is realized by normalizing the XANES spectra by their variance and fixing the first row of **T** to , where *u*_1_ and σ_1PC_ are the first PC and the corresponding singular value, respectively.^67^ In our case, we obtained *a* = – 0.32. We further notice that the XANES spectra should be nonnegative, and that the maximal physically reasonable value of each spectrum (the W.L. amplitude) is also limited. Here, we assume that it should be lower than 1.5, a value slightly larger than the maximum of the W.L. of the initial state XANES spectrum. We note here that higher W.L. amplitudes are not observed in experimental Ni K-edge data for any of the relevant reference materials, nor are they observed in XANES spectra simulations for relevant structure models (vide infra). It is possible to identify now the area of feasible solutions (AFS) containing all those **T** matrix elements satisfying these constraints. Considering that both aforementioned spectra-specific constraints for each species depend only on two **T** matrix elements (since each column of the matrix **T** affects just one of the spectra in matrix **S**), the determination of the corresponding AFSs is a straightforward linear 2D problem that can be solved by linear programming methods. These AFSs are shown as blue polygons in Figure 4. One can see, nonetheless, that while the spectra-specific constraints limit significantly the possible values of the **T** matrix elements, the solution associated with the spectral decomposition is far from being unique, requiring to impose additional constraints. To this aim, we note that the concentration values in the matrix **C** should be characterized by numbers between 0 and 1. Since the matrix **C** depends on the inverse of matrix **T**, the concentration values are functions of all 6 unknown elements of matrix **T**, requiring us to look for feasible solutions in a 6-dimensional (6D) space. Here we relied on a brute-force approach, where we constructed 6D vectors by randomly sampling points from the three (identical) two-dimensional AFS regions, identified in the previous step, and checked whether the resulting 6D vector satisfy the concentration constraints.

We also note here that swapping the rows in matrix **T** would result in a formally different but physically identical solution (therefore, the dark blue polygons in Figure 4a–c have identical shapes). To remove the ambiguity associated with the permutation of the spectra of pure species within matrix **S**, we postulate that the species that we labeled as the “first”, “second”, and “third” ones should have, respectively, the largest, second largest, and the smallest contribution to the third experimental XANES spectrum in our dataset (i.e., the spectrum collected after ca. 18 min under CO_2_RR). Here, we have used the third XANES spectrum because we expect all three species to be significantly contributing to this signal. The AFSs narrowed down based on these constraints and conventions are marked with light blue points in Figure 4a–c. As one can see, there is still some ambiguity in our solution to the spectral decomposition problem. As an illustration, in Figure 4d, we show three representative examples of possible XANES spectra for the second component satisfying the aforementioned constraints. This ambiguity requires us to make additional assumptions. Among the possible solutions, we highlight a particular one, where the first and the last spectra in the experimental operando XANES dataset **X** correspond to pure species, rather than to a mixture of different chemical components. These solutions correspond to an intuitively reasonable situation, where the structure of singly dispersed, homogeneous catalysts is fully transformed under reaction conditions, but the transformation proceeds through an intermediate step, where an additional transient species is present. Such a scenario is, in part, implied by our PCA results (see Figure S15). In Section 3.4, we will confirm this hypothesis by XANES simulation results.

In situations where the first and last spectra cannot be considered as corresponding to pure species, other additional constraints need to be introduced. Some spectral profiles can be fixed as corresponding to some known reference compounds. Alternatively, imposing constraints on the concentration profiles (based, e.g., on some kinetic models) can also reduce the ambiguity of the result. Furthermore, in some cases, it is possible to complement the XAS dataset with additional spectra collected under different conditions (e.g., under different applied potentials, pH values, etc.) in order to enhance the appearance of some species contributing to the heterogeneous structure of the catalyst.^38^

**Figure 5:**
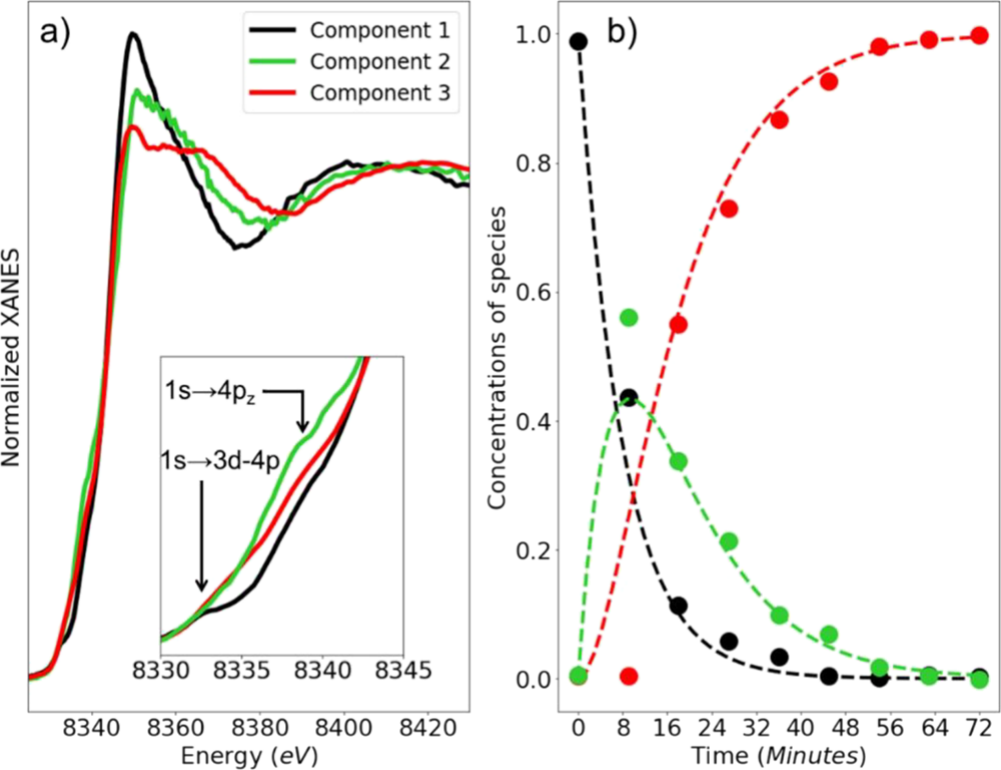
(a) XANES spectra for the extracted pure species and (b) related concentration profiles (filled circles) extracted via TM approach from the experimental Ni K-edge XANES data for the HT Ni-TMNC sample. The inset in (a) shows a magnification of the corresponding XANES pre-edge region. The dashed lines in panel (b) represent the fits of the obtained concentration profiles with a simple model of consecutive first-order reactions model.

This additional assumption of the purity of the first and last spectra in our matrix **X** leads to a single-point solution, indicated by the red crosses in Figure 4a–c. The final transformation matrix can be written as . The corresponding spectra and concentrations profiles for these pure species are shown in Figure 5. The obtained concentration profiles appear to be consistent with a simple kinetic three-component model (consecutive first-order chemical reactions): ; ; . Here, τ_1→2_ and τ_2→3_ are characteristic times (inverse rate constants) for the transformations of the first species into the second and of the second species into the third, respectively. By solving the differential equation system and fitting the obtained concentration profiles, we obtain τ_1→2_ = 1.0 min and τ_2→3_ = 1.4 min. Overall, the retrieved concentration profiles follow reasonably this simple kinetic model, thus confirming also the validity of all the physical/chemical assumptions described above. We note here that the values of the characteristic times τ_1→2_ and τ_2→3_ are much smaller than the time resolution of our experiment (as indicated in Section 2.4, ca. 9 min). The limited experimental time resolution results in the ambiguity of the exact time values that should be associated with each datapoint in Figure 5b, This could explain why the concentration profile for the intermediate species at *t* = 8 min deviates from the predictions of our kinetic model. To confirm (or disprove) the validity of such a simple kinetic model for this process, a more detailed analysis involving XAS data collected with time resolution better than 1 min would thus be necessary. Such an analysis, however, is outside the scope of this article.

### 3.4 Machine Learning-Assisted Identification of the Structures of the Pure Species

After isolating the XANES spectra for the pure species via a TM approach, one still needs to deduce the corresponding structures of these three species. Previous works have demonstrated that ab initio XANES simulations are well suited for the task of structure determination in single site catalysts.^7,13^ However, the effect of possible variations of a large number of relevant local structural parameters (e.g., distances between atoms of different types, bonding angles, etc.) on XANES spectra need to be considered in such analysis. Taking into account that the XANES simulations are computationally demanding, the direct fitting of XANES spectra is thus challenging. Instead, for the interpretation of the obtained Ni K-edge XANES profiles, we employed a supervised machine learning (SML)-based XANES fitting approach, as implemented in the PyFitIt code. Using this methodology, we first established a non-linear relation between the local structural parameters for the Ni species and the corresponding XANES profiles. For this purpose, we relied on relatively small training sets of ab initio XANES spectra (from 200 to 1000 spectra) obtained using the FDMNES code.^68,69^ We highlight here that the FDMNES code is able to successfully reproduce XANES spectra of relevant reference compounds such as the Ni K-edge XANES spectrum of the NiO compound, see Section S16 of the SI text, which gives us confidence in the reliability of these simulations for our purposes.

The set of structural parameters for which we performed XANES calculations was determined based on an Adaptive Sampling approach (active learning).^70^ This sampling scheme allows us to ensure higher density of calculated spectra in the regions of the structural parameters space, where small variations in structure parameters could result in large variations of the corresponding XANES spectra. Thus, the total number of required XANES calculations is minimized (see Figure 7a).^71^

We used the structural parameters sets and the respective calculated XANES spectra as the training datasets for an SML algorithm. Here, the objective is to construct a mathematical model μ̂(*E*; ***p***) that is a function of the energy *E* and a set of the corresponding structural parameters ***p***. μ̂(*E*; ***p***) must be able to interpolate between the points in the training datasets and thus to provide XANES spectra for those structural parameters ***p*** for which the explicit XANES calculations have not been carried out. For this purpose, we relied on a radial basis function algorithm (RBF). Here, the SML model is constructed as a linear combination of a set of basis functions: , where *N* is the number of calculated spectra in the training set, *K*(*r*) is a linear radial basis function, and *P_E_*(***p***) is a second-order polynomial depending on ***p*** with energy-dependent coefficients. We obtained then the unknown factors *w_i_* and the polynomial coefficients by requesting that the model should describe the XANES spectra in the training data set as closely as possible. To this end, *w_i_* and the polynomial coefficients were obtained through the least squares and ridge regression methods, respectively.^30,51,71^ We tested the accuracy of the trained SML routine using 10-fold cross-validation. Herein, the training set was divided randomly into 10 parts, nine of which were used for the algorithm training, while the last one for the validation. In accordance to the PyFitIt convention, the SML accuracy is then defined as , where the summation is carried out over all the points in the validation data set. μ_*i*_ is the spectrum calculated by FMDNES for the *i*th combination of parameters ***p***_*i*_, μ̂_*i*_ is the approximated spectrum yielded by SML, while *M* is the average XANES spectrum evaluated over all the spectra composing the training set. In all cases discussed below, we achieved an accuracy value higher than 0.98, indicating that our SML yields spectra closely match those obtained using the exact FDMNES simulations. The obtained SML routine can then be used to efficiently generate XANES spectra for different sets of structure parameters, and thus allows us the direct fitting of XANES spectra for the pure species identified by the TM.

**Figure 6:**
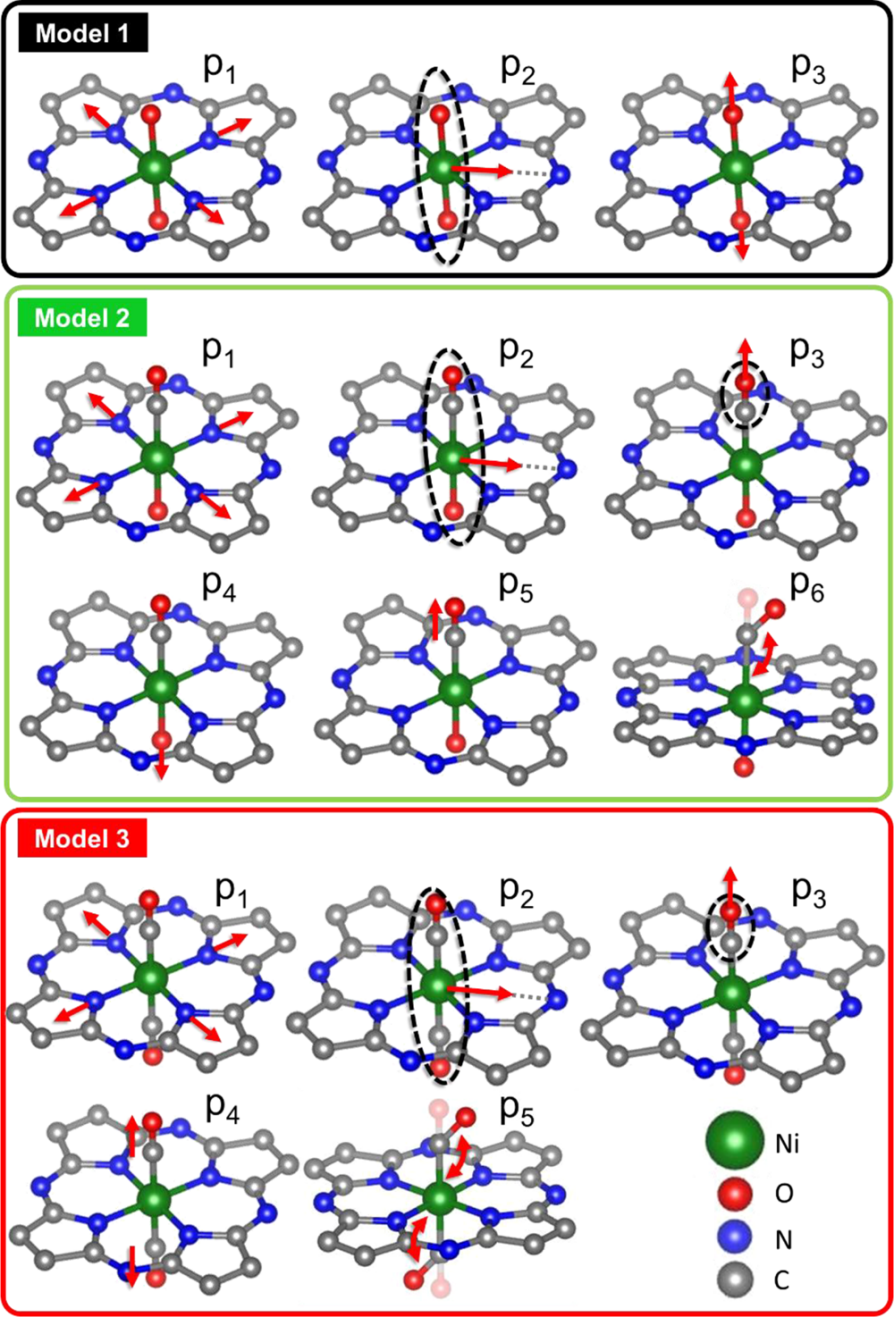
Set of possible deformations applied to the structure models used for the construction of the SML training data set and XANES fitting.

Based on the qualitative analysis of the XANES data and on the metal single sites models proposed in the prior literature for TMNC catalysts for the CO_2_RR^2,6,72,73^ and ORR,^7,13,74^ we focus on several distinct families of possible candidate structures for different states of our HT-Ni-TMNC sample. First, as discussed above, our qualitative analysis of the XANES data suggested that in the as-prepared state, Ni appears to be octahedrally coordinated, with a local structure resembling that of nickel oxide. Therefore, we considered a model where a Ni atom is surrounded by four pyridine ligands sited at 1.95 Å,^75^ with two additional axial oxygen atoms added at a distance of 2.0 Å from the Ni absorber (see model 1 in Figure 6). We expect this model to be representative of the structure of our as-prepared sample. Furthermore, our qualitative analysis suggested that under CO_2_RR conditions, the catalyst experiences significant changes in the local structure, but the singly dispersed cationic nature of Ni species is preserved. We have found that the replacement of one (model 2 in Figure 6) or both (model 3 in Figure 6) axial O atoms with a CO ligand in the afore discussed model leads to XANES spectra resembling the determined spectra for the second and third pure components, respectively. Here we keep the guessed C–O distance close to 1.1 Å. We used these structural models with varied interatomic distances and angles to construct the training data set and, consequently, to establish the corresponding interpolating functions μ̂(*E*; ***p***). The parameters that were allowed to vary and the ranges of their possible values are shown in Figure 6 and listed in Table 1.

**Table 1:** List of Structural Parameters for the Models Shown in Figure 6 Employed in the Fit of the XANES Spectra for Pure Components

parameter	description	range of variation
MODEL 1 (COMPONENT 1)		
p_1_	contraction/expansion of the pyridine ring	[−0.2: +0.2] Å
p_2_	shift of the Ni atom and the two O atoms toward the edge of the pyridine ring	[0: +0.3] Å
p_3_	contraction/expansion of the two Ni–O bonds	[−0.2: +0.2] Å
MODEL 2 (COMPONENT 2)		
p_1_	contraction/expansion of the pyridine ring	[−0.2: +0.2] Å
p_2_	shift of the Ni atom, the O atom, and the CO group toward the edge of the pyridine ring	[0: +0.3] Å
p_3_	contraction/expansion of the axial Ni–C bond	[−0.2: +0.2] Å
p_4_	contraction/expansion of the Ni–O bond	[−0.2: +0.2] Å
p_5_	contraction/expansion of the C–O bond	[−0.2: +0.2] Å
p_6_	Ni–C–O bond angle	[135: 180] °
MODEL 3 (COMPONENT 3)		
p_1_	contraction/expansion of the pyridine ring	[−0.2: +0.2] Å
p_2_	shift of the Ni atom, and of the CO groups toward the edge of the pyridine ring	[0: +0.3] Å
p_3_	contraction/expansion of the axial Ni–C bonds	[−0.1: +0.2] Å
p_4_	contraction/expansion of the C–O bonds	[−0.2: +0.2] Å
p_5_	Ni–C–O bond angle	[135: 180]°

We have also considered other possible distortions of the structure models, such as the shift of the Ni atom out of the pyridine ring plane, as suggested by refs (2, 6, 22, 72−74, 76, 77), see Figure S18 and Table S5. However, we found that such distortions do not agree with the observed W.L. and post-edge XANES features in our spectra (see Section S8 of the SI). In addition, we tried also to quantify and classify the importance of the different chosen structural parameters on the XANES spectra introducing a normalized standard deviation estimator,^30^ as explained in Section S9 and Table S8. From the obtained results one can see that the largest variations of the XANES spectra are associated with the stretching of the pyridine ring and with the changes in the Ni–O and Ni–CO bond lengths. Smaller, but not negligible, changes in the XANES spectra are also caused by the in-plane displacement of the Ni atoms and by the variations in the C–O distance. Less pronounced effects on the XANES spectra are caused by the change of the Ni – C – O angle, while the rotation of the CO group around the C–Ni–C axis, analogous to that suggested for the Cu-CO sites in ref (78), in turn, did not affect the calculated XANES spectra significantly and we thus concluded that our approach is insensitive to this parameter (see Section S9 of the SI text) and we thus neglected it in the fit.

**Figure 7:**
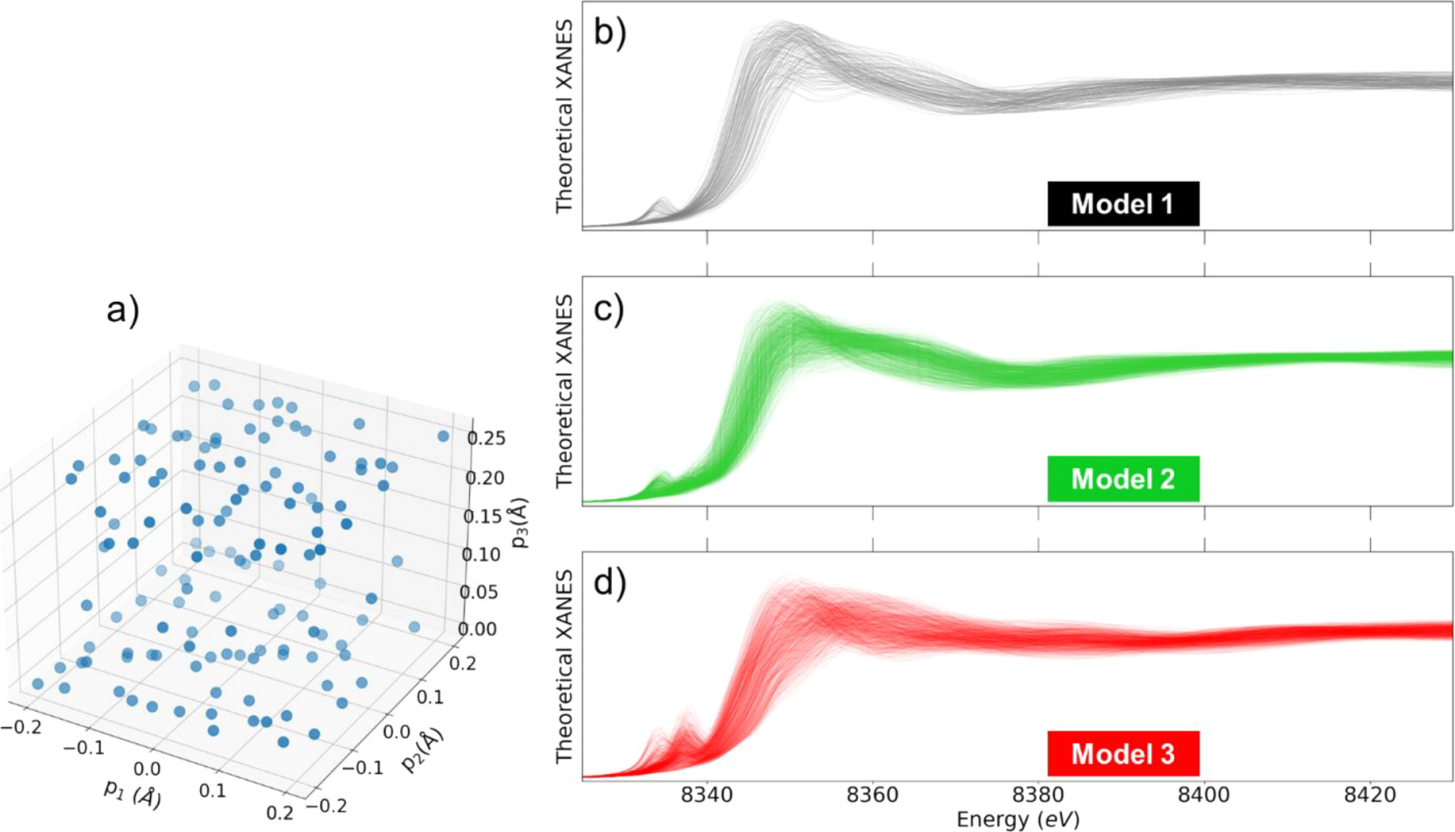
(a) Points in a structural parameter space obtained using the adaptive sampling employed to establish the μ̂(*E*; ***p***) interpolating functions for model 1 depicted in Figure 6. (b) Calculated spectra for the structure parameters corresponding to the points indicated in (a). (c, d) Representative calculated spectra for models 2 and 3 are shown in Figure 6.

**Figure 8:**
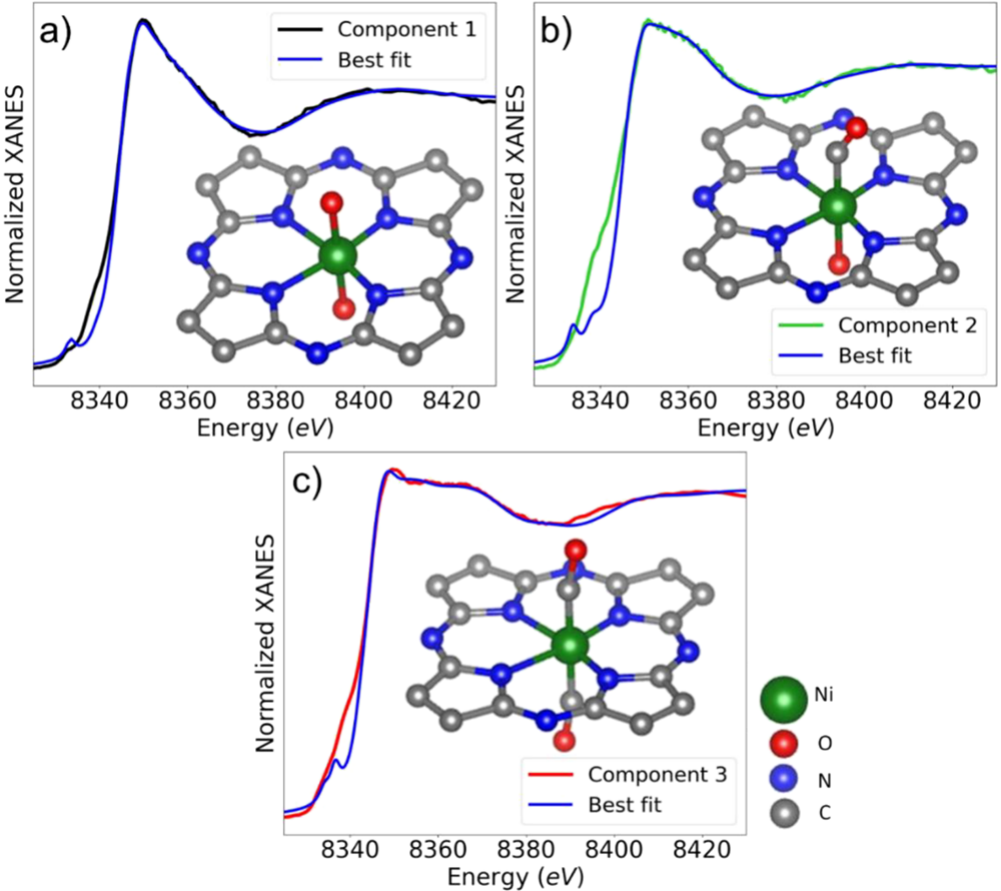
Comparison of the XANES components for pure species, as extracted from the experimental data, with the best-fit results. Insets show the final structure models obtained in the XANES fitting.

For the discussed sets of structure models, we carry out XANES simulations (Figure 7b–d), train the interpolating functions, and employ them to fit the XANES components derived by the TM method from the experimental data. Technical details concerning the choice of the FDMNES simulations parameters and parameters of spectra convolution, as well as more details about the XANES fitting procedure are given in Sections 6 and 7 of the SI text. The final best-fit results are shown in Figure 8.

Tables 2 and S10 report the final refined structural parameters for all structure models, in particular, average distances between Ni and its nearest neighbors plus the Ni–CO ligand bond angle.

**Table 2:** Interatomic Distances and Ni–CO Ligand Angles, Calculated for the Final Structure Models Obtained through the XANES Fitting Shown in Figure 8^a^

bond/angle	XANES best-fit value
MODEL 1 (COMPONENT 1). Misfit (*F*_min_): 0.02%	
Ni–N (two pyridinic N atoms closer to Ni)	1.75 (2) Å
Ni–N (two more distant pyridinic N atoms)	2.03 (2) Å
Ni–O	2.00 (2) Å
MODEL 2 (COMPONENT 2). Misfit (*F*_min_): 0.02%	
Ni–C (C of the CO group)	1.91 (3) Å
Ni–N (two pyridinic N atoms closer to Ni)	1.69 (2) Å
Ni–N (two more distant pyridinic N atoms)	1.97 (2) Å
Ni–O	2.07 (4) Å
C–O (C and O of the CO group)	1.04 (6) Å
Ni – C – Ô bond angle	149 (5)°
MODEL 3 (COMPONENT 3). Misfit (*F*_min_): 0.02%	
Ni–C (C of the CO group)	1.78 (3) Å
Ni–N (two pyridinic N atoms closer to Ni)	1.82 (3) Å
Ni–N (two more distant pyridinic N atoms)	2.17 (3) Å
C–O (C and O of the CO group)	1.27 (4) Å
Ni – C – Ô bond angle	170 (5)°

a The uncertainties of the last digit are shown in parentheses. They are derived from the ones shown in Table S10. For the details of the misfit quantity (*F*_min_) calculations, see Section S7 of the SI text.

To demonstrate that the obtained solution is well within the region where our constructed interpolating functions are accurate, in Figure S25, we compared the XANES spectra, obtained by using the constructed interpolation functions, with the spectra directly calculated by FDMNES for the final structure models.

### 3.5 Validation of the Structural Models via Reverse Monte Carlo EXAFS Analysis

To check whether the structure models derived based on XANES data fitting are consistent also with the available EXAFS data, we perform reverse Monte Carlo (RMC) simulations.^52^ In the RMC-EXAFS approach, we start with a 3D structure model obtained using the XANES fitting procedure and slightly move the atoms in the model around their initial positions in a random process in order to include the thermal and static disorder effects. Thus, we achieve the best possible agreement between the experimental Ni K-edge EXAFS data and the theoretical EXAFS spectra calculated for the given structure model. Note that the sensitivity of EXAFS to disorder effects is much higher than that of XANES. While these effects can be largely neglected in the XANES modeling, they need to be taken into account in the interpretation of the EXAFS data. The maximal allowed atom displacements from their starting positions in our RMC simulations are 0.4 Å; thus, the overall 3D structure of the material and coordination numbers do not change in the RMC-EXAFS fit. The RMC approach allows us to fit EXAFS data and account explicitly for the contributions of distant coordination shells, multiple scattering effects, as well as for the non-Gaussian shapes of bond-length distributions. Thus, we can fully benefit from all the information, encoded in EXAFS data, and can reliably fit the EXAFS data for strongly disordered materials such as TMNCs. All of these factors make RMC well suited for the interpretation of EXAFS data in TMNC catalysts, providing that the initial structure model is available.

To fit the EXAFS spectrum for the as-prepared HT-Ni-TMNC, we use as an initial structure the model obtained from XANES analysis (Figure 8a). To fit the EXAFS spectrum for the final state of the HT-Ni-TMNC catalyst under CO_2_RR conditions, we start with the structure model as shown in Figure 8c. Considering that a single structure model, as depicted in Figure 8, contains only one absorbing Ni atom, while the experimental EXAFS spectra are averaged over a large number of Ni species, to properly describe the bond length distributions the structure models that are optimized in our RMC-EXAFS simulations consist of 64 replicas of the models shown in Figure 8, placed at sufficiently large distances from each other. A similar approach was previously used by us to fit the EXAFS spectra of small oxide^39^ and metal^79^ nanoparticles. RMC fits of the EXAFS spectra are performed in *k* and *R*-space simultaneously, using wavelet transform to compare experimental and theoretically simulated EXAFS spectra. The refs (39, 41) provide more details on RMC-EXAFS simulations.

**Figure 9:**
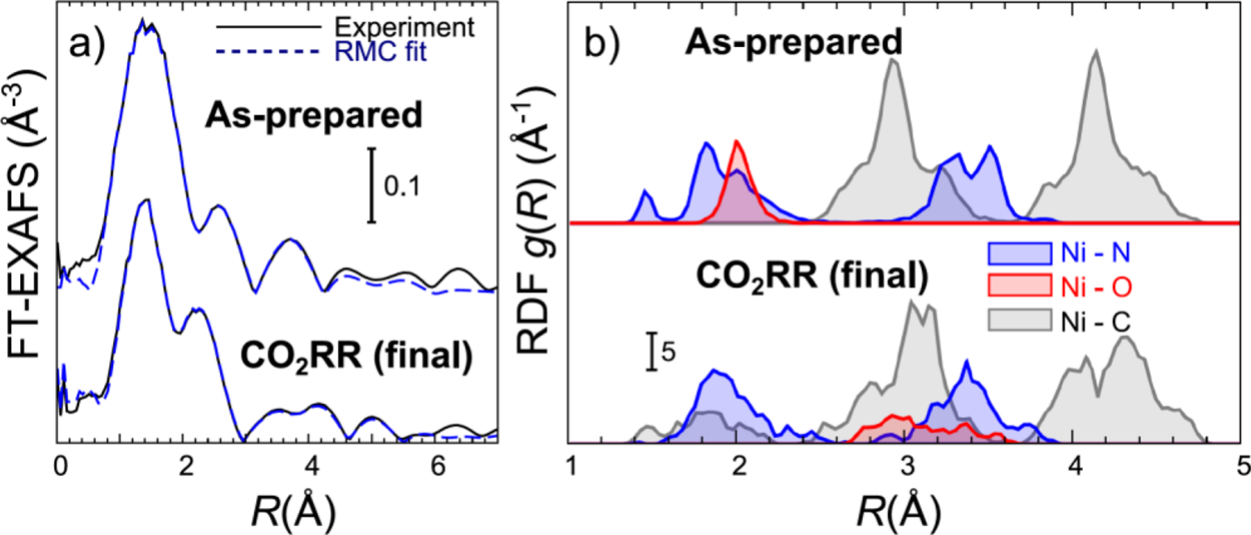
(a) Results of the RMC-EXAFS simulations. Comparison of Fourier-transformed experimental Ni K-edge EXAFS spectra for as-prepared HT-Ni-TMNC and for HT-Ni-TMNC catalysts in the final state under CO_2_RR conditions with the corresponding theoretical EXAFS data, calculated for the final structure models obtained in RMC simulations. (b) RDFs calculated from the atomic coordinates in the final RMC models. Partial RDFs for Ni–N, Ni–O, and Ni–C atoms are shown. FT-EXAFS spectra and RDFs are shifted vertically for clarity. Fourier Transforms in (a) are carried out in the *k*-range between 3 and 10 Å^–1^. RMC fits are carried out in *k*- and *R*-spaces simultaneously using wavelet transform, in the *k*-range between 3 and 10 Å^–1^ and the *R*-range between 1 and 6 Å, including multiple scattering contributions with up to 5 backscattering atoms.

Figures 9 and S27 show the RMC fits for the as-prepared HT-Ni-TMNC catalyst and HT-Ni-TMNC catalyst in its final state under CO_2_RR conditions. RMC simulations yield structure models that are in an excellent agreement with the experimental EXAFS data, both in wavelet space (Figure S27) and in *R*-space (Figure 9a). Thus, this confirms that the structure models obtained from XANES fitting do not contradict the available experimental EXAFS data. The structures obtained from the RMC-EXAFS fits are best analyzed in terms of radial distribution of atoms of different types around the central Ni atom. Figure 9b shows such partial radial distribution functions (RDFs) both, for the as-prepared HT-Ni-TMNC catalyst and the same catalyst in its final state under CO_2_RR conditions. The broad, split shapes of the RDF peaks indicate the strongly disordered structure of the HT-Ni-TMNC material. In particular, we note that all RDF peaks of Ni–N contribution in the as-prepared sample are split, suggesting that the Ni atom is located at an off-center position with respect to the square defined by 4 nearest N atoms, in agreement with the conclusions from XANES fitting. Furthermore, in our RMC-EXAFS results for the as-prepared sample, the average Ni–O bond length is similar to that of the longest Ni–N bond, and both are ca. 2.0 Å long, which is in excellent agreement with our results from XANES data fitting (Table 2). For the sample under CO_2_RR, the width of the Ni–N RDF peaks is similar to that in the as-prepared sample, but the individual subpeaks cannot be resolved, suggesting a further increase in the structural disorder under reaction conditions. The shortest Ni–C bond, associated with the interactions between Ni and CO adsorbates, results in a very broad RDF peak between 1.4 and 2.2 Å, with the maximum at ca. 1.6–1.8 Å, in excellent agreement with the Ni–CO bond length obtained in the XANES fit (ca. 1.78 Å). Note, however, that due to a very large disorder factor, the contribution of this bond to the experimental EXAFS spectrum is very small, and thus, the sensitivity of EXAFS to the presence of this bond is expected to be low.

## 4 Discussion

Focusing on Figures 8 and 9, we conclude that both the simulated XANES and EXAFS spectra for the final structure models agree well with the available experimental data. This gives us confidence that the atomistic structure models, constructed based on the TM method and XANES fitting, are representative of the structures of the as-prepared catalysts, intermediate state, and the final state achieved under CO_2_RR conditions. In the case of the XANES analysis, a particularly good agreement between the experimental and simulated spectra is observed in the post-edge region. A reasonable agreement between the XANES simulations and the extracted spectra for the pure species is achieved also in the pre-edge region, despite the fact that the approximations employed in the FDMNES code are known to be less accurate in this region.^30,80^

Moreover, as evident from our RMC-EXAFS results, the structures around each Ni site are flexible, resulting in large structural disorder. By attempting to reproduce the experimental XANES data with a single structural model, the structural disorder effect is neglected. This could affect significantly the relative intensities of the pre-edge features. The correct sampling of these deviations in the XANES modeling would require ab initio molecular dynamics or Metropolis Monte Carlo approaches,^81^ which are outside the scope of this article. Here, we just note that, statistically, there could be some configurations characterized by one Ni–O or Ni–CO ligand located closer to the Ni site, i.e., with a more pyramidal geometry characterized by a more intense 1s → 4p_z_ pre-edge feature.

**Figure 10:**
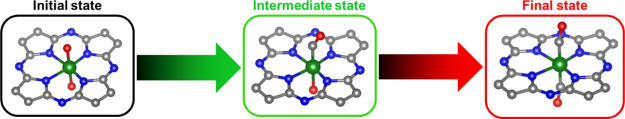
Main reaction steps suggested on the basis of the PCA and the XANES and EXAFS fitting results.

Finally, combining the results emerging from the PCA, TM method, and the XANES and EXAFS analyses, it is possible to propose the mechanism of the Ni speciation during the CO_2_RR reaction as depicted in Figure 10.

The initial state, before the reaction starts, is represented by a Ni site in a nearly octahedral coordination, where Ni is coordinated with two axial O atoms at ca. 2 Å (belonging, e.g., to two O atoms from the adsorbed water molecules) and shifted from the pyridines ring center by 0.2 Å. During CO_2_RR, one O atom appears to be replaced by a CO ligand, deriving from the activation of the CO_2_ molecule on the Ni center. We note that spectra very similar to our Ni K-edge spectrum for the intermediate state were reported in the literature for the [Fe(tren(py)_3_)]^2+^ ground state^82,83^ (Fe K-edge XANES) and for Co-based TMNC complexes (Co K-edge XANES).^84^ These Fe K-edge and Co K-edge XANES spectra in the prior works were attributed to octahedrally coordinated species, resembling the structure model suggested by our XANES fit. For this intermediate state, we have found that the position of Ni with respect to the pyridine ring is similar to that in the as-prepared state, while the ring itself is slightly more contracted (*p*_1_ = – 0.09 Å). The length of the Ni–O bond is increased to 2.07 Å, while the Ni–CO bond length is found to be ca. 1.91 Å, with a Ni – C – Ô angle of 149° and with a C–O distance of 1.04 Å.

The intermediate state is consequently converted into the final state through the substitution of the remaining O atom with an additional CO ligand. The fit of the final state with a geometry possessing a Ni site with a lower coordination number (i.e., with none or just one CO group) did not result in a good agreement with the experimental data (Section S8).

The XANES fitting results for the final catalyst state suggest an expansion of the pyridine ring of +0.07 Å, a larger shift of the Ni site outside the ring center and the presence of two CO groups sited at a distance of 1.78 Å from the Ni site. In this configuration, the first coordination shell for Ni effectively consists of four atoms: two N and two O. The remaining two N atoms are found to be located at larger distances (ca. 2.17 Å). The XANES analysis indicates also that the Ni–C–O angle is ca. 170°. On the other hand, looking at the map of correlations between different variables (Figure S26), it is evident that all the Ni–C–O angle values in the range between the 165° and 175° could also provide a good agreement with the experimental data. In particular, a nearly collinear Ni–C–O configuration with just slightly expanded average C–O distance could also fit well the XANES spectrum provided by the TM method.

The shift of the Ni site inside the pyridines ring deserves a special mention. As shown in Section S9, we found this parameter to be highly relevant for obtaining a good fit with the experimental data. The off-center displacement of Ni is, in fact, necessary to reproduce properly the W.L. and post edge regions for all three spectral components identified by the TM-XANES approach. RMC-EXAFS approach reinforces this finding yielding a very broad distribution of Ni–N bonds for the working catalyst, suggesting, again, a strong off-center displacement of Ni. The symmetry breaking in this case is likely explained by the presence of defects in the carbon support. Indeed, both experimental observations^85^ and theoretical simulations^86^ suggest that Ni–N sites are not distributed uniformly over the carbon support, but are localized in the vicinity of the edges of graphene layers (e.g., next to the pores in the support). This breaks the bi-directional symmetry of the system, allowing the distortions of the Ni–N_4_ structural motif. The presence of nonsquare-like motifs and their importance in electrocatalytic activity were reported also for Fe–N–C catalysts, where the distorted geometry can be tracked by Mössbauer spectroscopy.^87^ We also note that such distortions in the M–N_4_ structural motif may appear in EXAFS spectra as a decrease in the effective M–N coordination number. And, indeed, several recent EXAFS-based works on M–N–C catalysts suggest that the presence of metal sites with apparently reduced M–N coordination numbers could be decisive for the CO_2_RR activity.^24,59,88−92^

Overall, the scheme shown in Figure 10 with the gradual replacement of O or OH axial ligands by CO adsorbates under working conditions matches well the one recently reported for molecular Co single site catalysts for photocatalytic CO_2_ reduction,^84^ suggesting that the mechanism proposed here could be common in single site catalysts.

One should note that according to our findings XANES spectra are extremely sensitive to the details of the Ni local environment. Indeed, even slight changes in the bond distances and bond angles result in significant variations in the XANES features. As a result, the final XANES spectra could change depending on the sample preparation and from one sample to another, even if the structure around the active Ni site remains qualitatively similar. To illustrate that, in Sections S14 and 15 of the SI we show the results stemming from an analogous XANES analysis carried out for a Ni-TMNC sample that, unlike the HT-Ni-TMNC sample, was not exposed to the high-temperature treatment. We note that the spectrum for the final state of the Ni-TMNC sample differs remarkably from the spectrum for the HT-Ni-TMNC. Nonetheless, the final structure, featuring the Ni–N_4_ site coordinated with two CO ligands, is similar in both cases. This explains the similarity in catalytic properties observed for both of these catalysts.

## 5 Conclusions

In this work, we provided the first quantitative analysis of the structural changes experienced by Ni-based TMNC catalysts under CO_2_RR conditions. Only through the combination of time-resolved operando XAS measurements, unsupervised and supervised machine learning approaches and simulation-based XANES and EXAFS data fitting, we were able to identify the structures of the initial catalysts and those of their intermediate and final states under reaction conditions, as well as to reconstruct the concentration profiles of the different Ni species. Our results confirm that the single Ni sites are the active species for the CO_2_RR, but also reveal their dynamic, heterogeneous nature and adaptation to the reaction conditions. In particular, our data suggest the direct influence of the interactions between the Ni site and CO adsorbates on the XANES and EXAFS spectra, allowing us to get insight into the reaction mechanism, and highlighting the importance of operando spectroscopic investigations. We believe that the approach developed here will be helpful for understanding the active states of other TMNC catalysts as well, including single atom catalysts based on different transition metals and could also be applied for the understanding of other reactions, such as the oxygen reduction reaction, the photocatalytic CO_2_ conversions,^93,94^ and many others where single metal sites are considered to be attractive catalytic motifs.

## Supporting Information Available

Details of cells used for electrocatalytic activity and operando XAS measurements, ex situ characterization of the catalysts and its electrocatalytic activity measurements, supplementary XANES and EXAFS data, results of wavelet transform analysis of EXAFS spectra; description of the principal component analysis, FDMNES simulations, XANES normalization and fitting; XANES fitting results with additional structure models; analysis of the importance of individual fitting parameters; validation of the accuracy of the employed machine learning method; details of the RMC-EXAFS simulations; XANES and EXAFS data for the Ni-TMNC catalyst and XANES fitting results for this catalyst (PDF)
