# Supplementary material for: Tracking the Evolution of Single-Atom Catalysts for the CO2 Electrocatalytic Reduction Using Operando X-ray Absorption Spectroscopy and Machine Learning
Source: J Am Chem Soc. 2023 Jul 31;145(31):17351–66. doi: 10.1021/jacs.3c04826 (PMC10416299; doi:10.1021/jacs.3c04826)
Supplement: Supplementary file 1 — ja3c04826_si_001.pdf [file ja3c04826_si_001.pdf]

Supporting information (SI)

Tracking the evolution of single atom catalysts for the CO_2 electrocatalytic reduction using *operando* X-ray absorption spectroscopy and machine learning

Andrea Martini, Dorottya Hursán[§], Janis Timoshenko[*], Martina Rüscher, Felix Haase, Clara Rettenmaier, Eduardo Ortega, Ane Etxebarria and Beatriz Roldan Cuenya[*]

Department of Interface Science, Fritz-Haber Institute of the Max Planck Society,14195 Berlin, Germany.

(§) Current address: *University of Szeged, Department of Physical Chemistry and Materials Science, Aradi sq. 1, Szeged, 6720, Hungary.*

(*) Corresponding Author: janis@fhi-berlin.mpg.de; roldan@fhi-berlin.mpg.de

Index

1 Cells used for the electrocatalytic activity and *operando* XAS measurements 2
2 *Ex situ* characterization and electrocatalytic activity measurements 3
3 XANES derivatives and EXAFS $k^2\chi(k)$ signals of the experimental dataset 7
4 Wavelet Transform analysis of the EXAFS spectra belonging to the initial and final state 7
5 Determination of the number of pure species using empirical tests 9
6 FDMNES simulation and convolution parameters 11
7 Normalization of the theoretical XANES and XANES fitting strategy 12
8 Machine learning-assisted XANES fit of the second and third XANES components using pure four and five coordinated models 13
9 Effect of the single parameter variations 16
10 Effect of the CO-group rotation around the Ni-C axis 18
11 Comparison between the ML-derived approximations and the exact FDM calculations 19
12 XANES best-fit results using models from Figure 8 of the main text 19
13 Reverse Monte Carlo simulations for the interpretation of Ni K-edge EXAFS data 20
14 XANES and EXAFS spectra of the Ni-TMNC catalyst initial and final states during CO_2RR 21
15 Machine learning-assisted XANES fit of the initial and final states of Ni-TMNC sample 21
16 References 24

1 Cells used for the electrocatalytic activity and *operando* XAS measurements

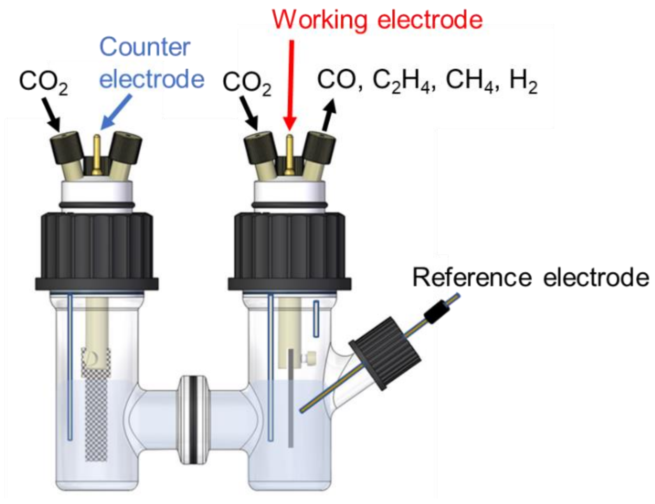

Figure S1: H-type cell scheme. RE: reference electrode, CE: counter electrode, WE: working electrode.

Figure S1 shows a schematic depiction of the H-type cell used for the catalytic activity measurements. This cell design allows CO_2RR product detection, while the semipermeable membrane (Selemion, AMV, AGC Inc.) that separates anode and cathode compartments and prohibits poisoning ions to pass towards the investigated electrode. The cathode compartment contains the WE and the RE while an inlet allows the CO_2 bubbling and an outlet leads the excess CO_2 and the gas products to the Gas Chromatograph. The anode compartment contains a counter-electrode (Pt gauze (MaTecK, 3600 mesh cm^{-2})) and an inlet for CO_2 bubbling. The used electrolyte was 0.1M $KHCO_3$, purified with a cation exchange resin (Chelex 100 Resin, Bio-Rad) and pre-saturated with CO_2.

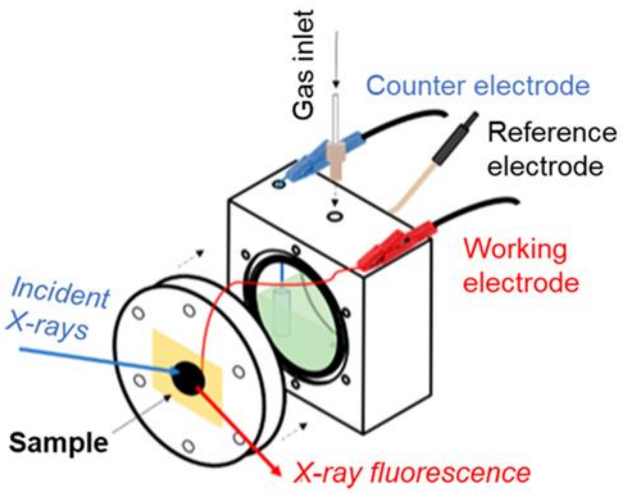

Figure S2: Operando electrochemical cell used to collect the XAS spectra during the CO_2RR conditions. Adapted with permission from Ref.[1] Copyright 2021 American Chemical Society.

The cell used for the *operando* XAS measurements is a single compartment cell with a typical three electrode setup featuring a reference electrode, counter electrode and, finally, a working electrode (our sample). The XAS measurements were performed in fluorescence mode mounting the sample on the front panel of the cell

facing the electrolyte and acting as an X-ray window. **Figure S2** shows the schematic depiction of the *operando* XAS cell.

2 *Ex situ* characterization and electrocatalytic activity measurements

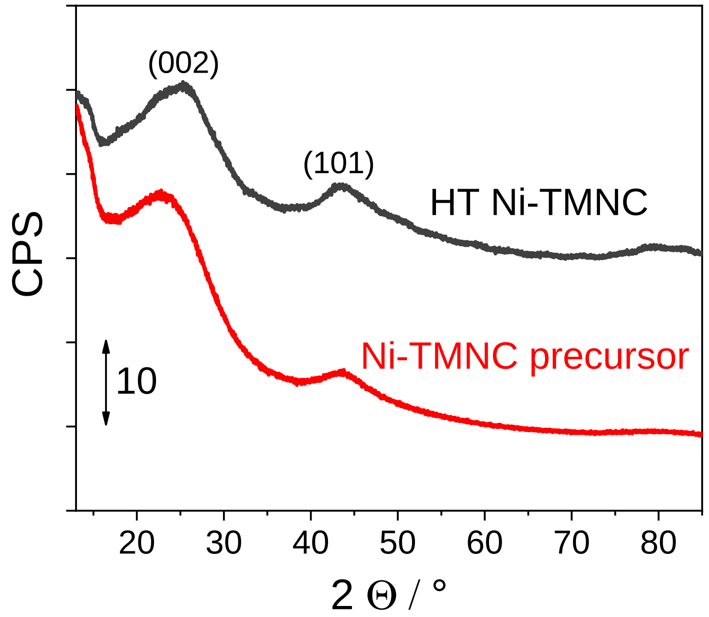

Figure S3: Powder X-ray diffractograms of the studied catalysts.

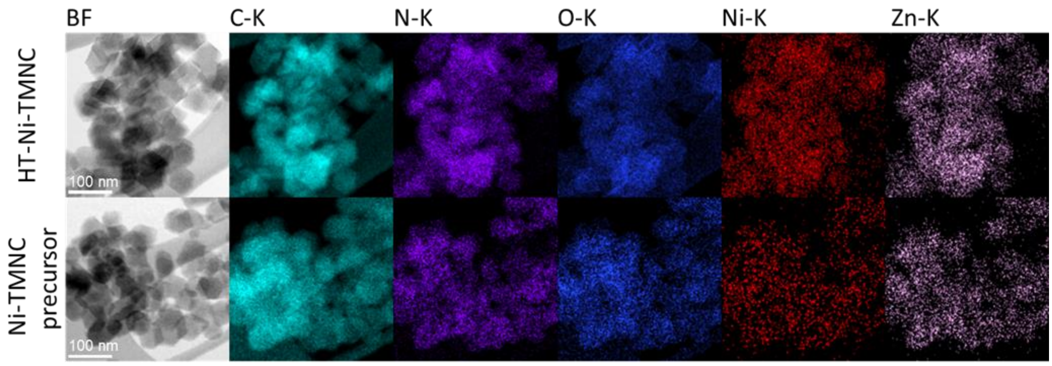

Figure S4: EDS mapping of the as prepared Ni-N-doped carbon catalysts highlighting the presence of Ni and remnant Zn.

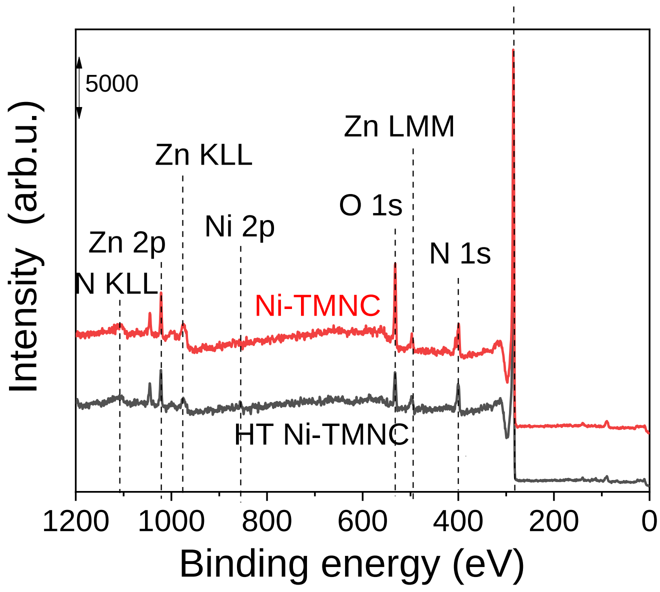

Figure S5: Survey X-ray photoelectron spectra of the two studied catalysts.

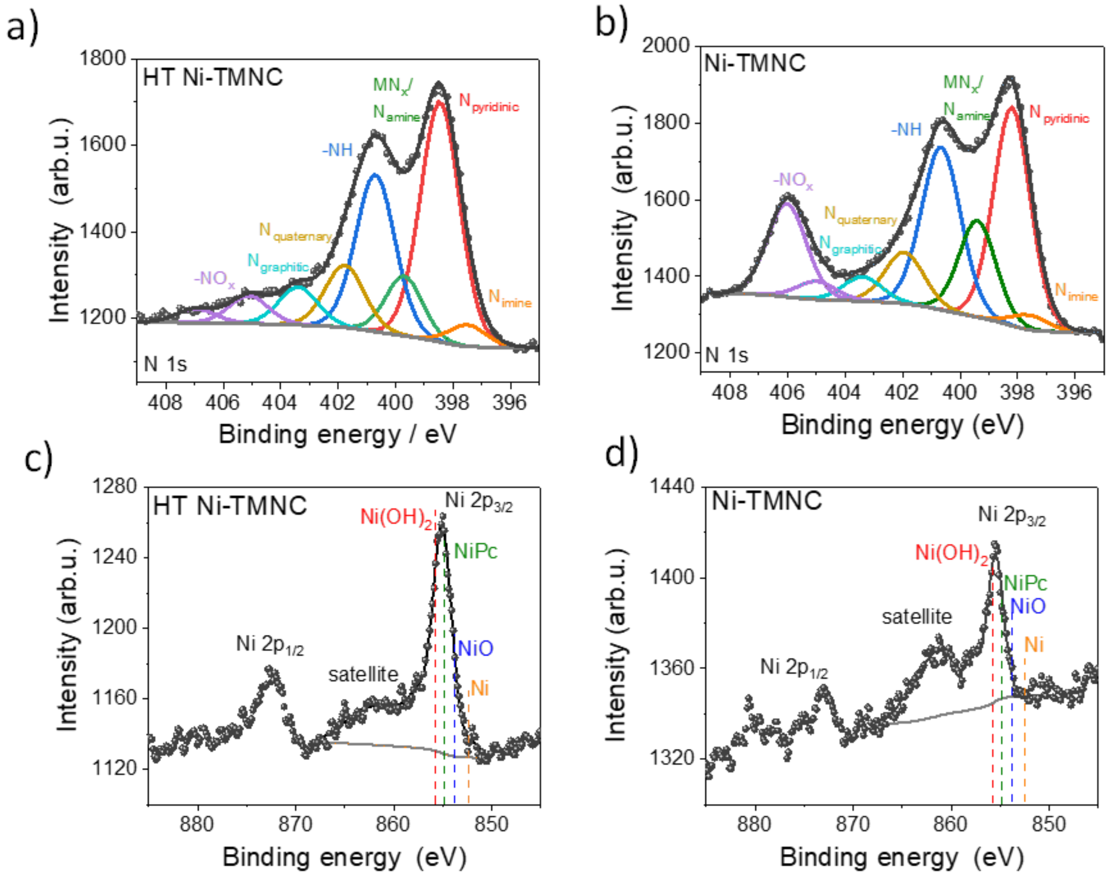

Figure S6: High resolution N 1s (a,b) and Ni 2p (c,d) regions of the X-ray photoelectron spectra of HT Ni-TMNC (a,c) and Ni-TMNC (b,d). In panels c and d the main line positions for reference Ni-compounds are shown which were taken from Refs.[2, 3]

The N 1s spectra were fitted with components in accordance with previous reports on transition metal nitrogen doped carbon (TMNC) materials.[4-9] The peak at 398.3 eV is assigned to pyridinic N (i.e., N bound to two

carbon atoms in a six-member ring). The peak located at 399.5 eV can be attributed to amine-type N or Nitrogen coordinated to Ni. The next peak at 400.8 eV is related to hydrogenated N-functional groups, which is either a pyrrolic-type N (i.e., N bound to two C and one H in a five-member ring) or a hydrogenated pyridinic N. We assign the peak centered at 402.1 eV to quaternary N (bearing a positive charge), and the peak at 403.5 eV to graphitic N. We also note that the latter two peaks may have contribution from protonated N-species as well, such as pyridinium ion. Finally, the two peaks between 405 and 407 eV are attributed to different oxidized N-moieties .[8]

Table S1: Relative surface chemical composition of the studied catalysts, determined from the XPS measurements.

	Rel. at.%					Ni/N ratio
**Catalyst**	**C**	**N**	**O**	**Ni**	**Zn**	
HT-Ni-TMNC	89.3	6.3	3.8	0.15	0.4	0.024
Ni-TMNC	85.9	6.9	6.6	0.10	0.4	0.014

Table S2: Metal-contents of the studied catalysts determined from ICP-MS measurements

Catalyst	**Ni**		**Zn**	
	**w%**	**at%**	**w%**	**at%**
HT-Ni-TMNC	0.70±0.07	0.14±0.01	1.9±0.2	0.35±0.04
Ni-TMNC	0.33±0.06	0.07±0.01	1.9±0.1	0.35±0.02

Table S3: Nitrogen speciation of the catalysts determined by the fitting of the high-resolution N 1s XPS spectra in **Figure S6**.

	Rel. at. %						
	**Imine N**	**Pyridinic N**	**MN_x/Amine N**	**N-H**	**Quaternary N**	**Graphitic N**	**$N-O_x$**
BE / eV	397.5	398.4	399.7	400.7	401.8	403.4	405-407
HT-Ni-TMNC	3.3	39.1	9.1	25.5	10.1	6.1	6.8
Ni-TMNC	2.1	31.1	13.8	24.7	7.8	3.7	16.9

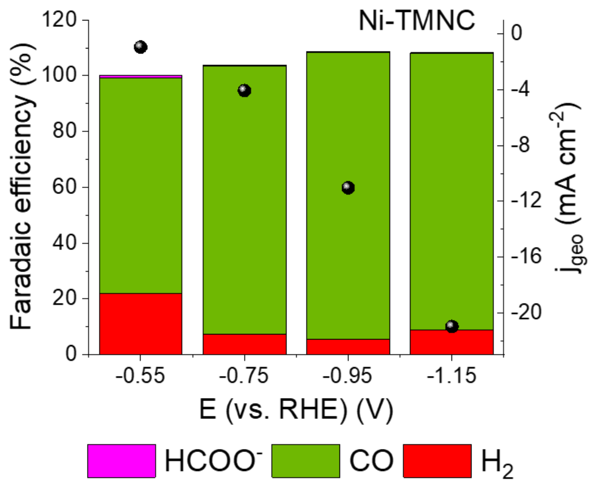

Figure S7: Catalytic selectivity of the Ni-TMNC catalyst in the CO_2RR: faradaic efficiencies and total current densities. Catalytic tests were performed under potential control in an H-type cell using a CO_2-saturated 0.1 M $KHCO_3$ electrolyte. Error bars give the standard deviation of the measured data for measurements performed on at least two separate electrodes.

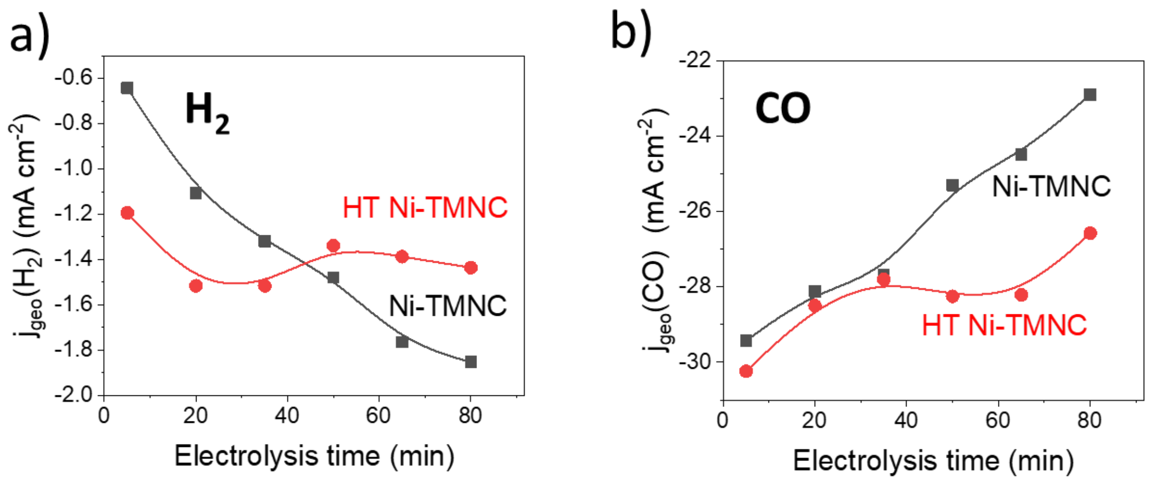

Figure S8: Partial current densities for (a) H_2 (a) and (b) CO on the studied catalyst over 5000 seconds of electrolysis at -1.15 V (vs. RHE) in 0.1 M $KHCO_3$ electrolyte. Lines serve only as a guide for the eye.

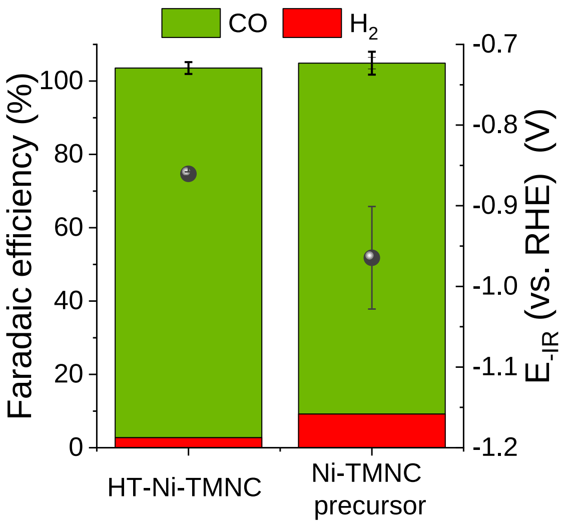

Figure S9: Catalytic selectivity of the Ni-TMNC catalysts in the CO_2RR: Catalytic tests were performed under current control with -15.7 mA cm^{-2} applied current density in an H-type cell using a CO_2-saturated 0.1 M $KHCO_3$ electrolyte. Error bars give the standard deviation of the measured data for measurements performed on at least two separate electrodes.

3 XANES derivatives and EXAFS $k^2\chi(k)$ signals of the experimental dataset

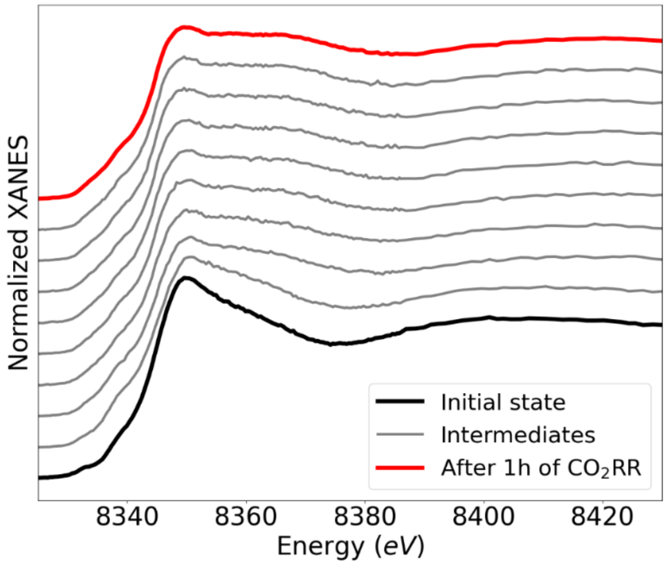

Figure S10: Ni K-edge (a) XANES and (b) magnitudes of the Fourier Transformed (FT) EXAFS (phase uncorrected) spectra collected during CO_2RR conditions for the HT-Ni-TMNC sample. The measurements were performed in a CO_2-saturated 0.1 M $KHCO_3$ electrolyte under a static current density of -15.7 mA cm^{-2}.

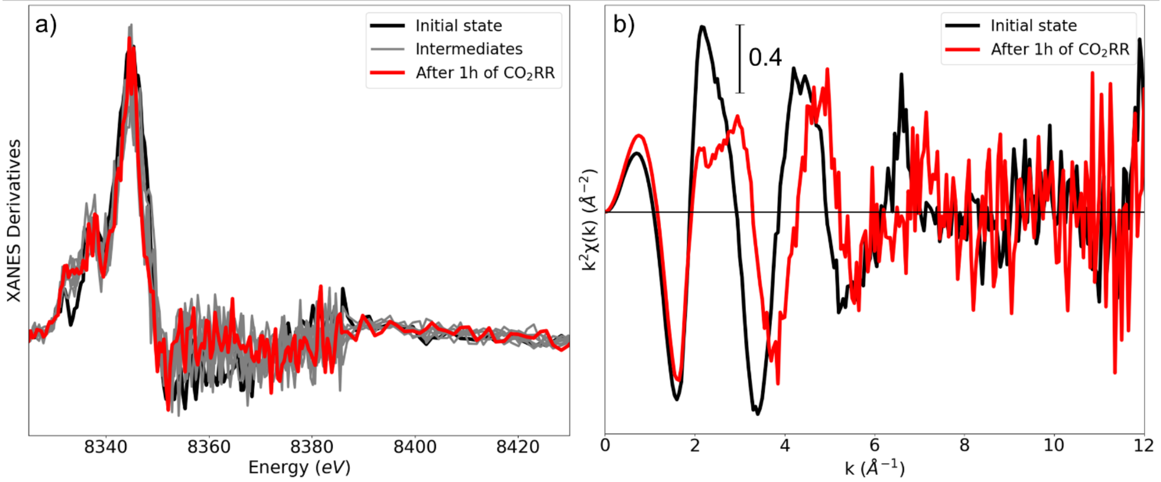

Figure S11: a) Derivatives of Ni K-edge XANES spectra for the as-prepared HT-Ni-TMNC before, during and after 1 h CO_2RR. b) Experimental Ni K-edge EXAFS spectra for the as-prepared HT-Ni-TMNC before and after CO_2RR. The measurements were performed in a CO_2-saturated 0.1 M $KHCO_3$ electrolyte under a static current density of -15.7 mA cm^{-2}.

4 Wavelet Transform analysis of the EXAFS spectra belonging to the initial and final state

To exclude the presence of possible Ni-Ni contributions, we relied on the wavelet transform (WT) analysis[10] of the EXAFS spectra involving the initial and final state of the reaction process. WTs of the EXAFS spectra for the initial and final catalyst state are compared with those for Ni metal and NiO XAS references spectra in **Figure S12**(a-d). The WTs were carried out using the Morlet mother wavelet function with $s = 1$ (value for the width of the Gaussian envelope), and $\eta = 7$ (frequency of the harmonic function), allowing the optimal resolution in *k*- and *R*- spaces for the WT-EXAFS features located at *R*-values around 3.5 Å. One can note that

the intensity of the contribution of particular scattering path to the WT-EXAFS map depends on the atomic number of the scattering element. Lighter elements typically show a maximum WT-EXAFS intensity at lower *k*-values than the heavy metals. Theoretical calculations show that the maximum of the Ni-Ni scattering amplitude function is expected to be at ca. 7Å^{-1} (**Figure S12**(f)). The WTs of the experimental Ni K-edge EXAFS spectra for metallic Ni and for Ni-Ni bond in NiO reference indeed exhibit a pronounced maximum in this region of *k*-space (**Figure S12**(a, b, e)). On the other hand, the maximum contribution of low-Z elements (N, C, O) is expected at ca. 3 Å^{-1} (**Figure S12**(f)), as exemplified by the Ni-O bond contribution of the WT-EXAFS map belonging to the NiO reference (**Figure S12**(b, e)). Here, to show it more clearly, in **Figure S12**(e) we plotted the WT-EXAFS data, integrated over R values between 0.5 and 4.0 Å: $\phi(k) = \int_{R_{min}}^{R_{max}} dR|WT(k,R)|^2$.[11, 12] We note that for our catalysts, both, in the as-prepared state and under CO_2RR conditions, only the maxima at lower *k*-values can be observed in the WT-EXAFS data (**Figure S12**(c, d, e)), suggesting that the Ni site is coordinated only with light backscattering atoms and allowing us to rule out the presence of significant amounts of metallic Ni clusters in the sample.

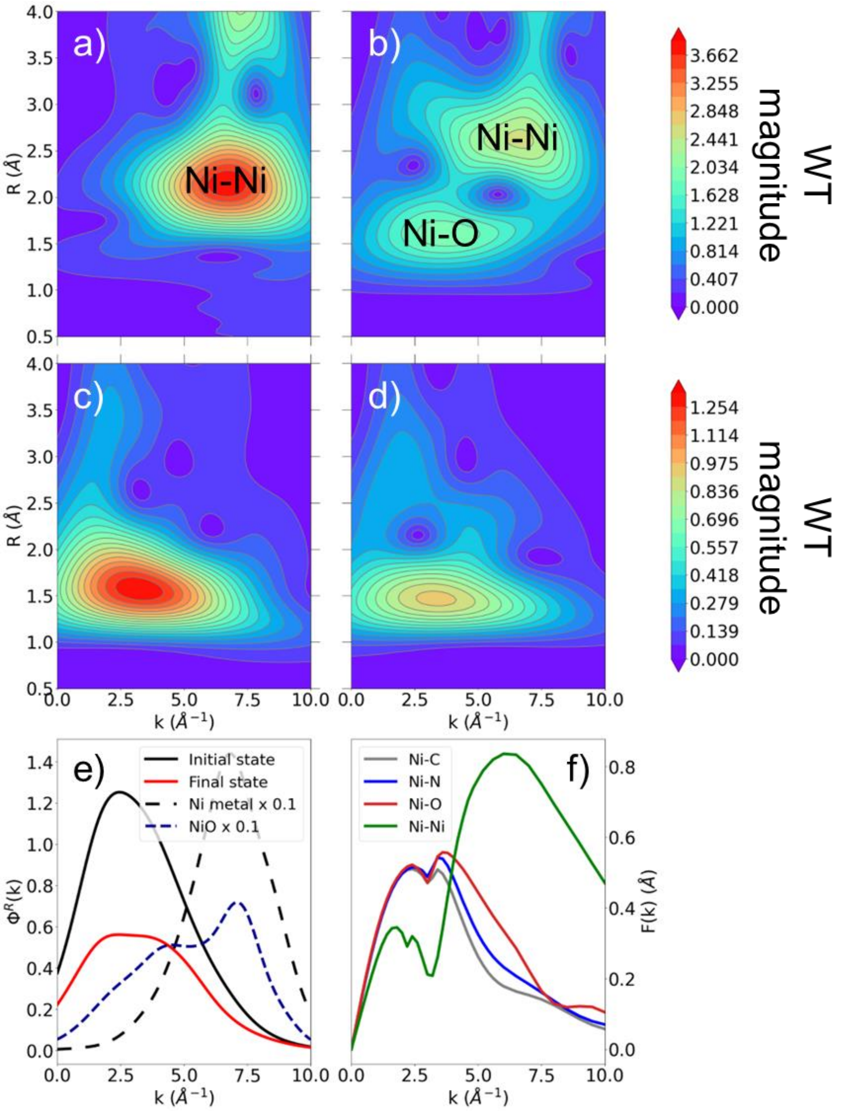

Figure S12: Wavelet transforms of Ni K-edge EXAFS data for (a) Ni metal and (b) NiO references. c-d) Wavelet transforms of EXAFS spectra for the HT-Ni-TMNC catalysts in the as-prepared and final states under CO_2RR conditions.

e) The intensity of WTs, integrated over all the R-values for the four WT representations reported in panels (a-d). f) Effective backscattering amplitude functions for Ni-C, Ni-N, Ni-O and Ni-Ni bonds calculated by FEFF code.[13] The measurements were performed in a CO_2-saturated 0.1 M $KHCO_3$ electrolyte under a static current density of -15.7 mA cm^{-2}.

5 Determination of the number of pure species using empirical tests

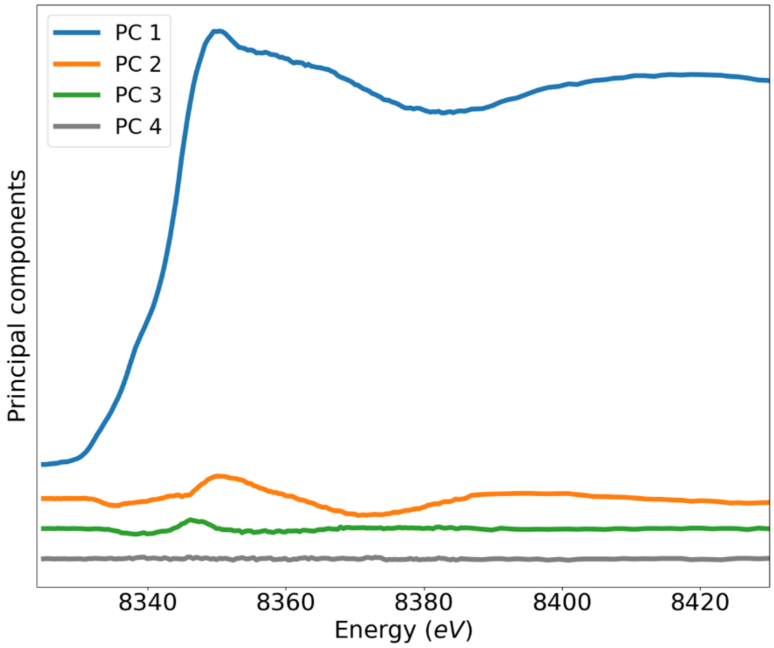

Figure S13: First four principal components determined from the set of operando Ni K-edge XANES data for the HT Ni-TMNC sample. The PCs are shifted vertically for clarity.

The PCA was carried out based on the singular value decomposition (SVD) of matrix **X** formed by experimentally collected XANES spectra.[14, 15] The first four extracted components are displayed in **Figure S13**.

To estimate the number of independent components, several statistical approaches could be used. In the Scree test (**Figure S14**(a)) one plots the sequence of singular values on a logarithmic scale. Herein we observe that all the singular values starting from the 4th one have similar values, thus they likely all correspond to experimental noise, and the number of pure species is equal to three.

On the other hand in the so-called IND test, the number of independent components is estimated by calculating following quantity: $\mathrm{IND}_v = \frac{1}{(n-v)^2}\sqrt{\frac{\sum_{i=v+1}^{n}\lambda_i}{m(n-v)}}$, with $\lambda_i = s_i/(m-1)$. Here v is the vth PC, s_i is the singular value associated to the $(v+1)^{th}$ component, n and m are the number of XANES spectra in the dataset and the number of points in each discretized spectrum. The IND factor is calculated increasing progressively the number of PCs considered until the minimum of the related IND_v quantity is found. The number of independent components corresponds to the minimum point of the empirical IND plots (**Figure S14**(b)). Similarly, in the imbedded error (IE) test (**Figure S14**(d)) one looks for the minimum of the quantity $\sqrt{\frac{v\sum_{i=v+1}^{n}\lambda_i}{mn(n-v)}}$. Both IND and IE tests confirms the presence of only three independent components.

Finally, the same result can be obtained also by finding the largest PC having a significance level (from the Fisher distribution) lower than 5% (**Figure S14**(c)) from the so called Malinowski's test plot.

As one can see, all these tests point to the same conclusion: three components are sufficient to describe the variations between XANES spectra in our dataset.

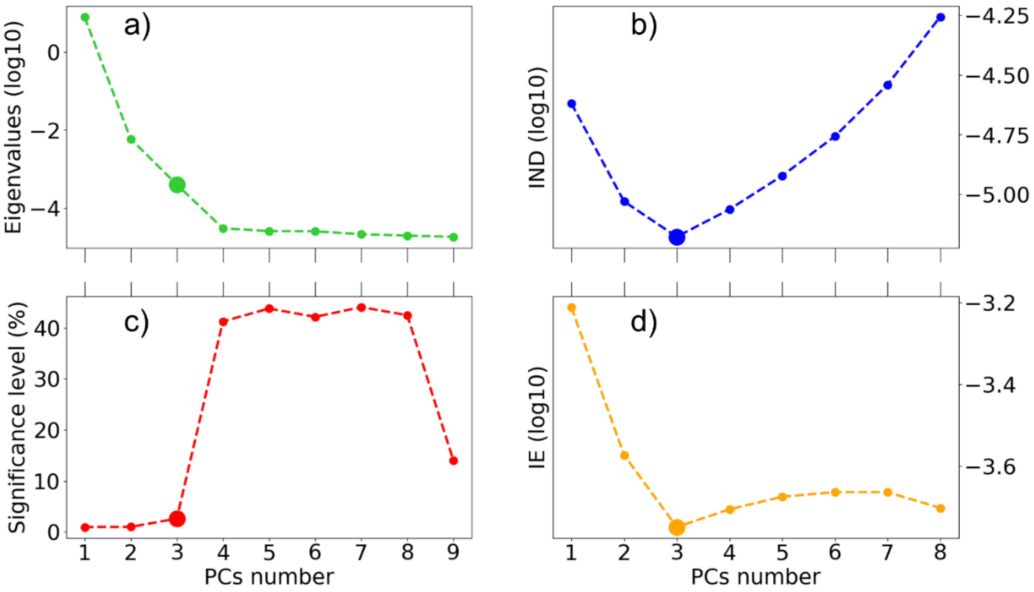

Figure S14: Empirical tests used to estimate number of pure species resulting in the variations between XANES spectra shown in **Figure 3** in the main text. a) Scree Test Plot. b) IND test Plot. c) Significance Level Plot (Fisher Test). d) Imbedded Error Plot (IE)

Additional information regarding the evolution of the three chemical compounds in the system can be achieved by analysing the residuals associated to the reconstruction of each XANES spectrum of the dataset involving two and three PCs. In particular, we introduce a lack of fit (% LOF) quantity, defined as: $\% \, \mathrm{LOF_i} = 100 \times \frac{||\mu_i(E) - \mu_i(E,v)||^2}{||\mu(E)||^2}$, where $\mu_i(E)$ is an i-th XANES spectrum of the dataset and $\mu_i(E,v)$ is the corresponding reconstruction obtained using the first v PCs. The $||\,.\,||^2$ operator denotes the squared L_2 norm operator.[16] The % LOF trends are depicted in **Figure S15**(a). One can see that using only two PCs the reconstruction error is the highest for the second/third XANES scan (corresponding to the state of the catalyst after ca. 9-18 minutes under CO_2RR). By employing three PCs in the XANES reconstruction, the lack of fit is decreased for these XANES spectra, **Figure S15**(b). We can thus conclude that all three Ni species contribute simultaneously only during a relatively short time interval of 9-18 minutes under CO_2RR, while the spectra corresponding to the catalyst in the initial and final state can be reconstructed using only two PCs. This suggests the presence of an intermediate transient species.

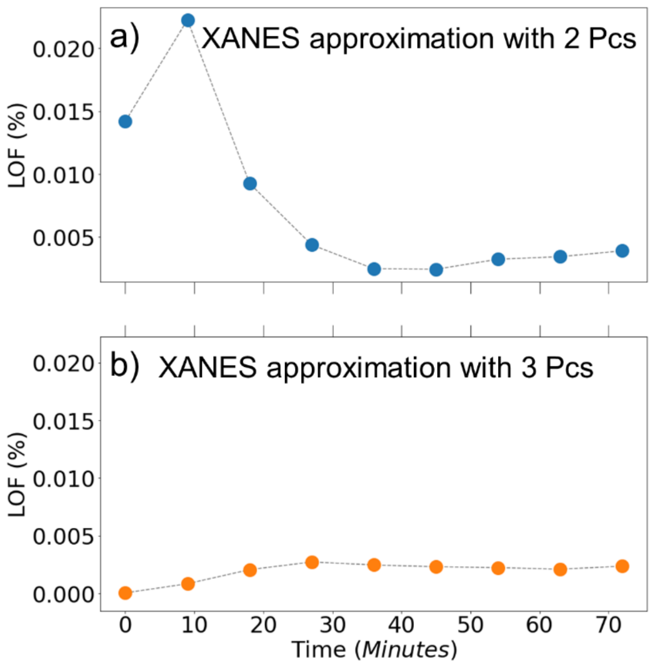

Figure S15: b, c) Lack of fit trend (LOF (%)) evaluated for each spectrum of the XANES dataset with two and three PCs, respectively. The LOF data are plotted as a function of time under CO_2RR.

6 FDMNES simulation and convolution parameters

For the Ni K-edge XANES simulations we relied on the finite difference approach (FDM), implemented in the FDMNES code.[17] The energy mesh used for spectra simulations featured an energy step of 0.02 eV near the Fermi level, while 2 eV and 30 eV above it. We performed the calculations using the real Hedin-Lundqvist [18] and von Barth local exchange correlation potential.[19] In the spherical region around the atoms and in the outer sphere, the electrostatic potential and the electron wave function were expanded in a series of spherical harmonics choosing the maximum value of the angular momentum l as $= \sqrt{l_{max}(l_{max}+1)}$, where k is the photoelectron wave vector and r is the radius of the sphere. The parameters reported in

Table S4 were selected for the convolution of the calculated spectra employing an energy dependent arctangent shape of the Lorentzian profile (details can be found in the manual for the FDMNES program[20]), see **Figure S16**(a).

The simulated spectra were aligned by correcting each energy grid by their related `EPSII` parameter (corresponding to the energy required to bring one 1s core electron to the continuum for each structure).[20] At the same time, we applied a common shift of 148 eV to all the simulated spectra. The latter was estimated following an approach introduced in Ref.[21] We performed FDMNES calculations for the Ni-phthalocyanine and aligned the maximum (`EPSII`–energy shifted) of partial density of *p*-states projected on the Ni absorber (PDOS), with the maximum of the white line (W.L.) intensity of the corresponding experimental Ni K-edge XANES, see **Figure S16**.

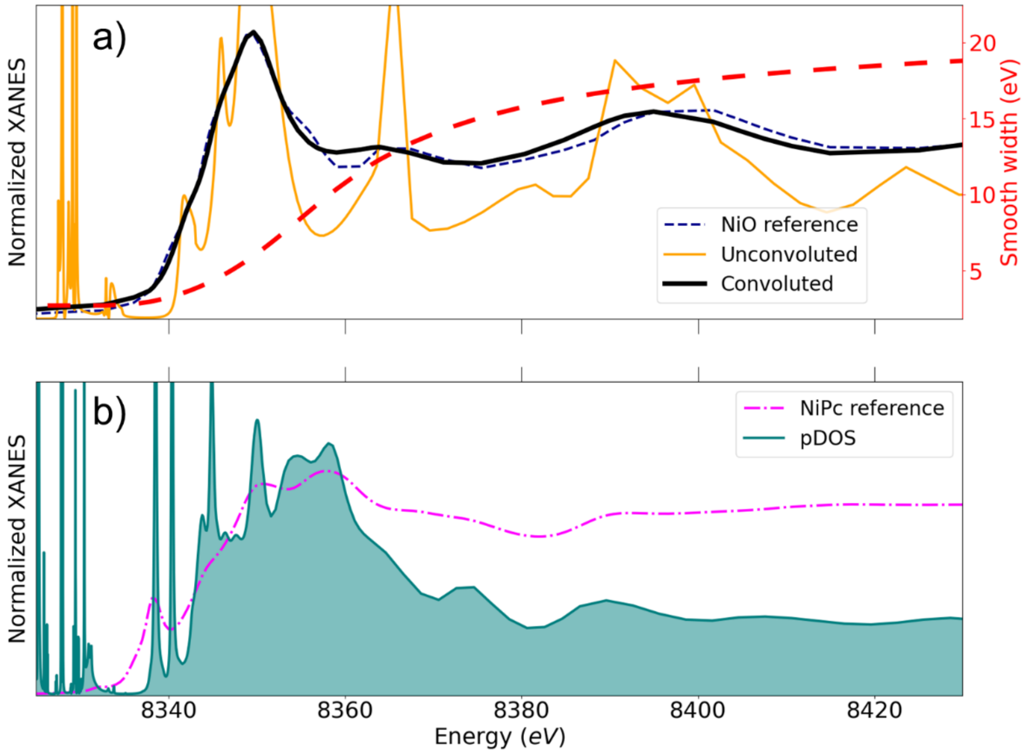

Figure S16: (a) Comparison of the experimental and theoretical Ni K-edge spectra. Theoretical spectra before and after convolution are shown, where the latter was obtained by convoluting the calculated spectra with the smooth edge step function (dash red curve) (b) Experimental Ni K-edge XANES for nickel pthalocyanine (reference) and the partial density of *p*-states simulated using FDMNES and projected on the Ni site (pDOS).

Table S4: Convolution parameters applied to all the simulated XANES spectra.

Gamma hole (eV)	Ecent (eV)	Elarge (eV)	Gamma max (eV)	E Fermi (eV)
2.70	31	36	19.40	8333.20

7 Normalization of the theoretical XANES and XANES fitting strategy

For comparison of calculated and experimental XANES spectra, we first normalized all the simulated spectra by a quantity α defined as: $\min_{\alpha}\|\mu^{exp}(E) - \alpha\mu_i\|^2$, where μ_i is a FDMNES simulated spectrum corresponding to the set of structure parameters p_i. Afterwards we trained the RBF algorithm on the sets of theoretical spectra to construct the interpolation functions. Finally, we fit the experimental XANES spectra using them. For the XANES fitting we used the coordinate descendent algorithm,[22] and we minimized the residual F-value quantity defined as: $F = \frac{\|\mu^{exp}(E) - \hat{\mu}(E;p)\|^2}{\|\mu^{exp}(E)\|^2}$. The latter was calculated in the energy range between 8342 and 8430 eV, excluding part of the XANES pre-edge region that is not perfectly reproduced using the FDM approach.[20] The lowest achieved F-value (denoted as F_{min}), can be used to characterize the goodness of fit and is reported in **Table 2** in the main text.

Since our experimental XANES spectra have a very good signal to noise ratio ($\sim 10^4$) the uncertainty associated with the obtained best-fit values for different structural parameters, is mostly associated with the correlations between different fitting parameters (as well as with the systematic e limitations of the theory or systematic errors in the experiment[23]). To estimate the uncertainties due to the correlations, we calculated the

degree of variations in the corresponding parameter value, which would result in an increase of the best fit-value F_{min} by 10%. More precisely, for each structural parameter p_i, we evaluated the F-value curve by changing p_i value within the range, specified in Table 1 in the main text, and optimising the values of remaining structural parameters. For example, **Figure S17** shows F-value curve for parameter p_5 (of model 3 (tilting of the Ni-C-O bond angle, see **Figure 6** of the main text). For comparison, we show also F-value curve, characterizing the changes in F-quantity, if the values of other structure parameters are not re-optimized after changing the value of p_5.

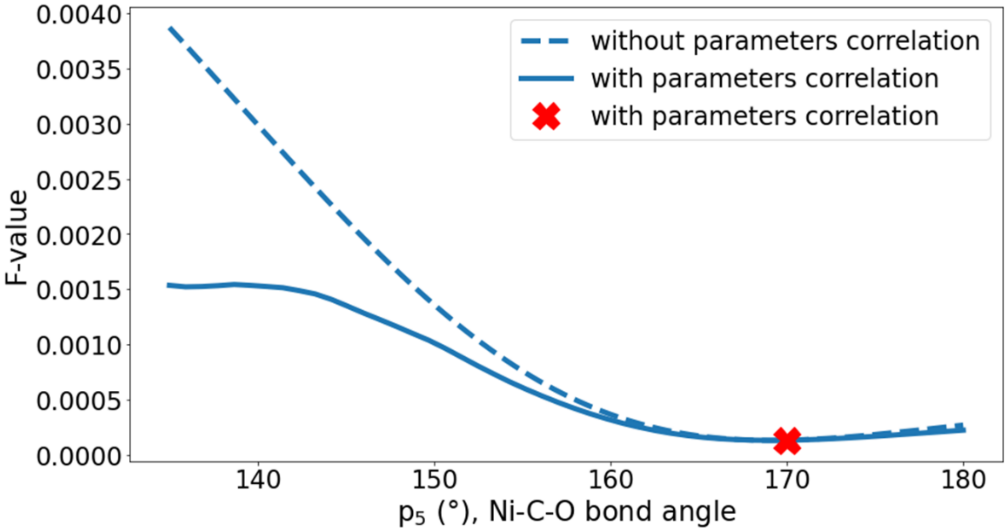

Figure S17: Error curve obtained for the parameter p_5 of model 1 referring to **Figure 6** of the main text The continuous line has been obtained by varying the parameter p_5 in the range between 135°-180° and optimising the remaining parameters (i.e., p_1-p_4). This approach allows to take into account the correlation effects of the chosen parameters and estimate the fit uncertainties. On the other hand, the dashed error curve was obtained by fixing the parameters p_1-p_4 to the best fit values and calculating the F-value while varying p_5 between 135°-180°.

8 Machine learning-assisted XANES fit of the second and third XANES components using pure four and five coordinated models

Figure S18 shows the two alternative tetrahedral (4- and 5-coordinated) structures employed in the fit of the intermediate (component 2) and final state (component 3) obtained through the TM methodology. As for the models shown in the main text, also here the training sets contained between 200 and 1000 theoretically generated XANES, which allowed to have a SML-accuracy value higher than 0.98, indicating a good level of approximation. In model 5 we set the initial value for the distance between the Ni and the CO ligand at 2.0 Å, as for the model 2 shown in **Figure 6** of the main text. At the same time, we employed the same structure for the fit of the XANES third component but setting the initial value of Ni-CO distance at 1.75 Å.

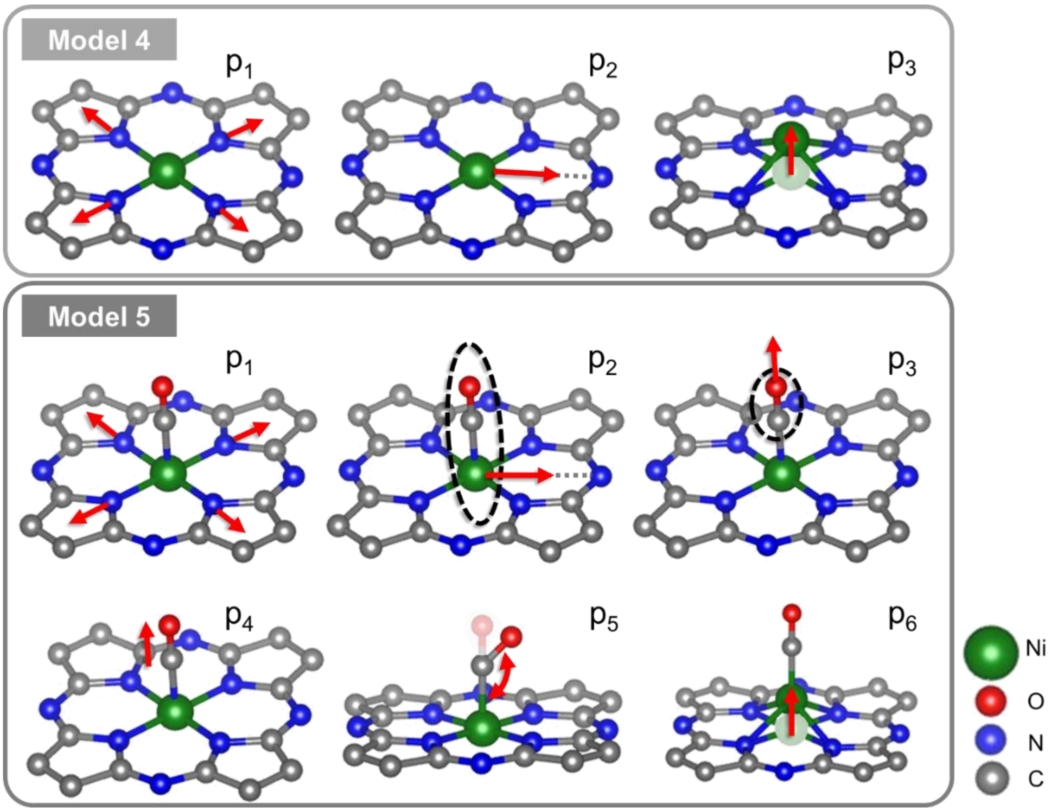

Figure S18: Set of deformations characterizing the four and five coordinated models used to fit additionally the second and third XANES components retrieved from the transformation matrix approach shown in **Figure 5** of the main text. The description of each parameter represented in this picture is summarized in **Table S5**.

Table S5: List of structural parameters shown in **Figure S18** and employed in the additional fit of the second and third XANES components of **Figure 6** of the main text.

Parameter	Description	Range of variation
	**Model 4 (Component 3)**	
p_1	Contraction/extension of the pyridine ring.	[-0.2 : +0.2] Å
p_2	Rigid shift of the Ni center towards the border of the pyridines ring.	[0 : +0.30] Å
p_3	Movement of the Ni atom outside the pyridine rings.	[0 : +0.3] Å
	**Model 5 (Component 2/Component 3)**	
p_1	Contraction/extension of the pyridine ring.	[-0.2/-0.1 : +0.2] Å
p_2	Rigid shift of the Ni center and of the CO group towards the border of the pyridine ring.	[0 : +0.3] Å
p_3	Contraction/extension of the CO ligand.	[-0.2 : +0.2] Å
p_4	Contraction/extension of the C-O distance.	[-0.2 : +0.2] Å
p_5	O tilting around the axis passing through the C atom.	[135 : 180] °
p_6	Rigid shift of the Ni atom and of the CO ligand outside the pyridines rings.	[0 : +0.3] Å

We employed the same normalization parameters adopted to weight the theoretical XANES spectra, deriving from the models shown in **Figure 6** of the main text, for the fit of the second and third component and of the precursor. In the following, we display the best-fit plots and the tables containing the refined parameters.

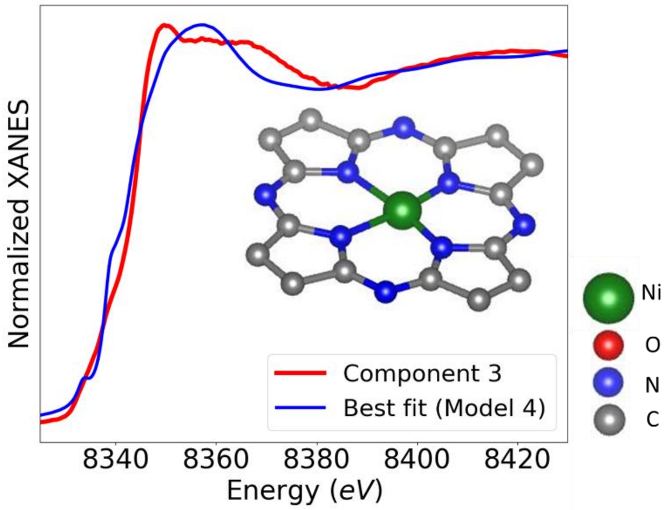

Figure S19: Comparison of the third XANES component (**Figure 5** of the main text) and its best-fit result obtained using the model 4 depicted in **Figure S18**. The inset shows the refined geometry. The refined structural parameters are reported in **Table S6** and **Table S7**.

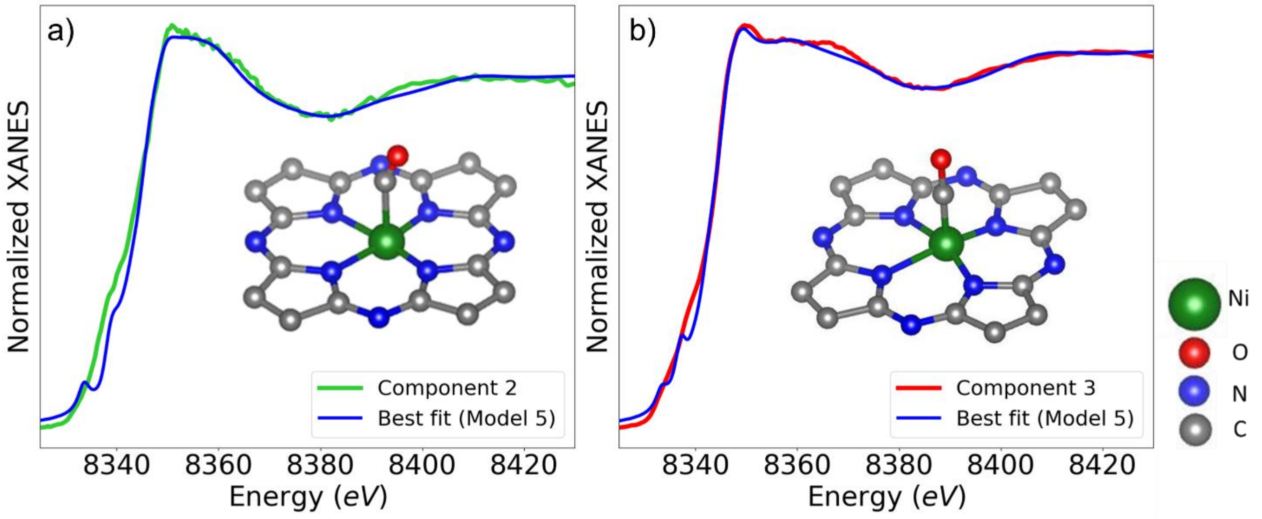

Figure S20: Comparison of the a) second and third b) XANES component (**Figure 5** of the main text) and the best-fit results obtained using the model 5 depicted in **Figure S18**. The insets show the refined geometries. The refined structural parameters are reported in **Table S6** and **Table S7**.

Table S6: Refined structural parameters obtained through the XANES fits shown in **Figure S19** and **Figure S20**.

Parameters	XANES Best Fit value
**Model 4 (Component 3)**	
p_1	-0.05(2) Å
p_2	0.20(3) Å
p_3	0.0(2) Å
**Model 5 (Component 2)**	
p_1	-0.07(2) Å
p_2	0.18(2) Å

p_3	0.03(2) Å
p_4	-0.20(3) Å
p_5	157(7) °
p_6	0.00(5) Å
**Model 5 (Component 3)**	
p_1	-0.02(4) Å
p_2	0.23(5) Å
p_3	0.02(5) Å
p_4	0.15(5) Å
p_5	165(9) °
p_6	0.3(2) Å

Table S7: Interatomic distances and Ni-CO ligand angles, calculated for the final structure models, obtained through the XANES fitting in **Figure S19** and **Figure S20**. The distances uncertainties are derived from the ones showed in **Table S6**. For the details of the misfit quantity (F_{min}) calculations see **Section S6.**

Distances (average)/Angle	XANES Best Fit value
**Model 4 (Component 3). Misfit (F_{min}):** 0.14 %	
Ni-N (two pyridines N atoms closer to Ni)	1.73(4) Å
Ni-N (two pyridines N atoms farer to Ni)	2.00(4) Å
Ni distance from the pyridines plane	0.0(2) Å
**Model 5 (Component 2). Misfit (F_{min}):** 0.02 %	
Ni-C (C of the CO group)	2.03(3) Å
Ni-N (two pyridines N atoms closer to Ni)	1.72(3)Å
Ni-N (two pyridines N atoms farer to Ni)	1.98(3) Å
C-O (C and O of the CO group)	0.90(3) Å
$\widehat{Ni - C} - O$ bond angle	157(7) °
**Model 5 (Component 3). Misfit (F_{min}):** 0.02 %	
Ni-C (C of the CO group)	1.77(5)
Ni-N (two pyridines N atoms closer to Ni)	1.76(6)
Ni-N (two pyridines N atoms farer to Ni)	2.07(6)
C-O (C and O of the CO group)	1.25(5)
$\widehat{Ni - C} - O$ bond angle	180(9) °

Although model 5 shows a more intense pre-edge peaks and comparable fitting quality in the pre-edge range as obtained with models 2 and 3, it provided worse agreement in the W.L. and in the post-edge region. These regions are where the FDMNES software should be more accurate.[17, 24]. Furthermore, the XANES fit using model 5 results in an unphysically short distance between C and O atoms in the CO group (0.90 Å).[25]

9 Effect of the single parameter variations

To characterize the effect of changes in different structural parameters on XANES spectra, we defined the quantity $\sigma_{norm}(p_i) = \frac{1}{E_{max}-E_{min}} \int_{E_{min}}^{E_{max}} dE \, \frac{\sigma[\mu(E;\, p_1,\dots,p_n)]_{p_i}}{\sigma[\mu(E;\, p_1,\dots,p_n)]_{p_1,\dots,p_n}}$, where p_i is the ith structural parameter and $\mu(E;\ p_1, \dots, p_n)$ is the theoretical XANES spectrum calculated by FDMNES for a given set of n structural parameters $\boldsymbol{p} = (p_1, \dots, p_n)$. $\sigma[\mu(E;\ p_1, \dots, p_n)]_{p_i}$. In the numerator, under the integral, is indicated the standard deviation of the XANES value with respect to the variation of the ith parameter p_i. This quantity is normalized by the analogous expression obtained by varying all the parameters within their range of allowed values. The obtained values of $\sigma_{norm}(p_i)$ for different structural parameters p_i are reported in **Table S8**.

Table S8: Normalized integral standard deviations calculated for the selected structural parameters assuming ten deformations for each parameter varying uniformly in the related range of definition.

	$\sigma_{norm}(p_1)$	$\sigma_{norm}(p_2)$	$\sigma_{norm}(p_3)$	$\sigma_{norm}(p_4)$	$\sigma_{norm}(p_5)$	$\sigma_{norm}(p_6)$
Model 1	1.26	0.42	0.85			
Model 2	1.78	0.52	0.65	0.55	0.48	0.36
Model 3	1.42	0.54	0.65	0.62	0.58	

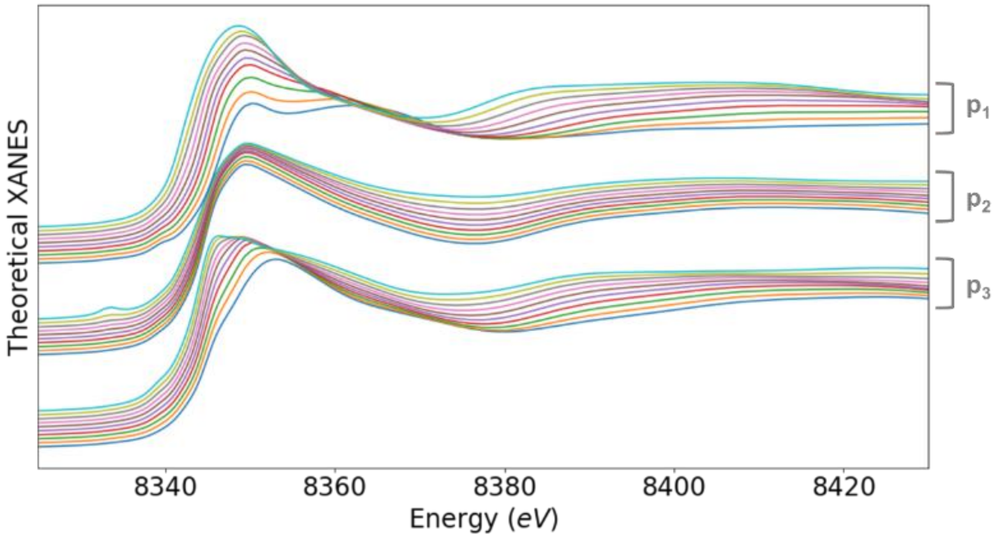

Figure S21: XANES changes associated to the linear variation of each single structural parameter belonging to model 1 within the ranges defined in **Table 1** and in **Figure 6** of the main text.

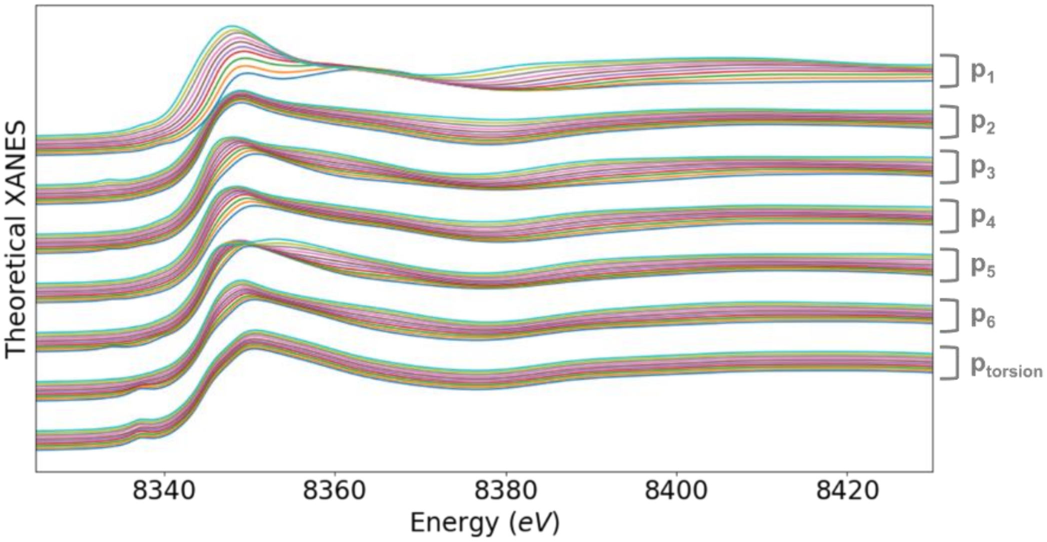

Figure S22: XANES changes associated to the linear variation of each single structural parameter belonging to model 2 within the ranges defined in **Table 1** and in **Figure 6** of the main text and in **Figure S24**(a).

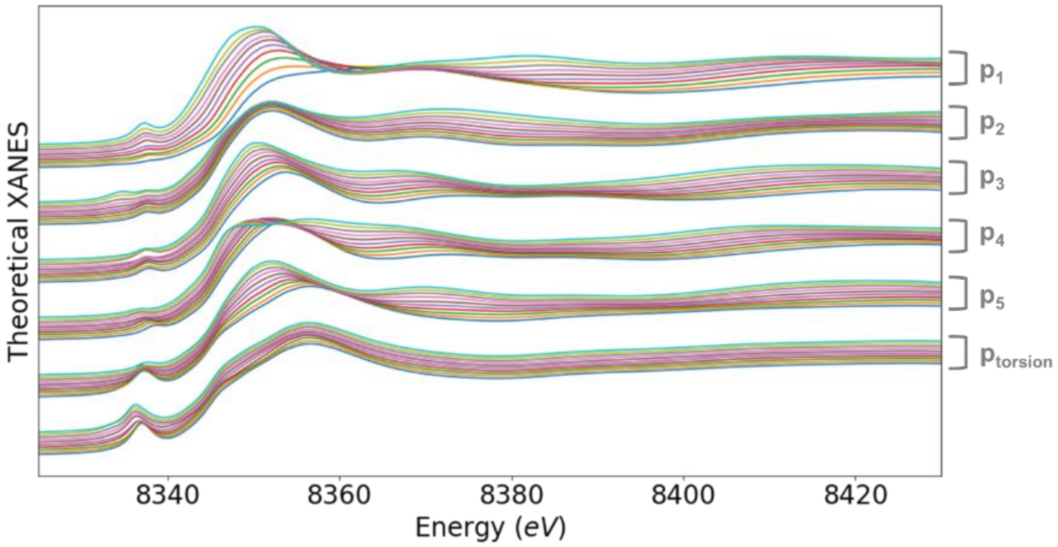

Figure S23: XANES changes associated to the linear variation of each single structural parameter belonging to model 3 within the ranges defined in **Table 1** and **Figure 6** of the main text and in **Figure S24**(b).

10 Effect of the CO-group rotation around the Ni-C axis

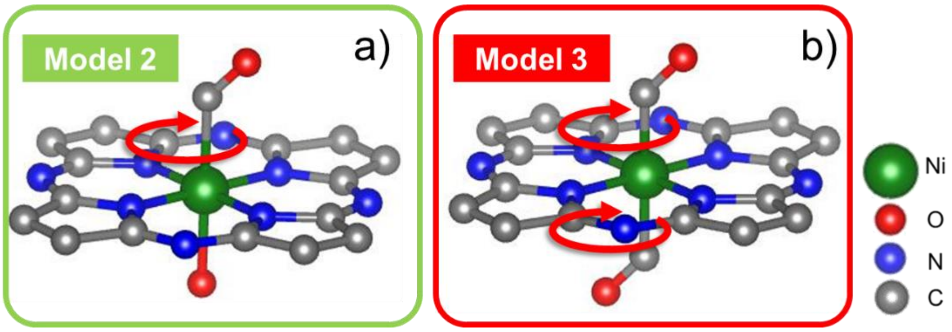

Figure S24: Graphical representation of the rotation of the CO group(s) around the Ni-C bond direction for the models associated to the second a) and third b) pure XANES spectra, respectively. Ten XANES spectra associated to these models assuming a fixed Ni-C-O angle of 45 ° are shown in **Figure S22** and **Figure S23**, respectively.

Table S9: Standard deviations associated to the rotations of the CO groups around the Ni-C bond axis for the two models (model 2 and mode 3), depicted in **Figure S24**, respectively.

	$\sigma_{norm}(p_{rot})$
Model 1	
Model 2	0.04
Model 3	0.14

Comparing these results with the standard deviations shown in **Table S8**, it is possible to see that their magnitudes are almost one order lower. This fact leads us to neglect this parameter in the XANES fitting.

11 Comparison between the ML-derived approximations and the exact FDM calculations

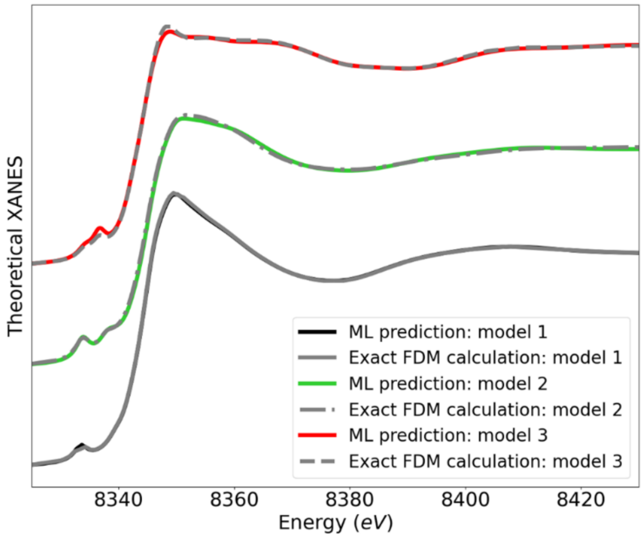

Figure S25: Comparison between the ML-predicted XANES spectra exact FDM calculations for the sets of structural parameters giving the best agreement with the XANES spectra for pure components.

12 XANES best-fit results using models from Figure 8 of the main text

Table S10: Refined structural parameters obtained through the XANES fitting procedure.

Fit variable	XANES Best Fit value
**Model 1 (Component 1)**	
p_1	-0.03(1) Å
p_2	0.20(2) Å
p_3	-0.00(2) Å
**Model 2 (Component 2)**	
p_1	-0.09(1) Å
p_2	0.20(2) Å
p_3	-0.09(3) Å
p_4	0.07(4) Å
p_5	-0.07(6) Å
p_6	149(5) °
**Model 3 (Component 3)**	
p_1	0.07(1) Å
p_2	0.25(3) Å
p_3	0.03(3) Å
p_4	0.17(4) Å
p_5	170(5) °

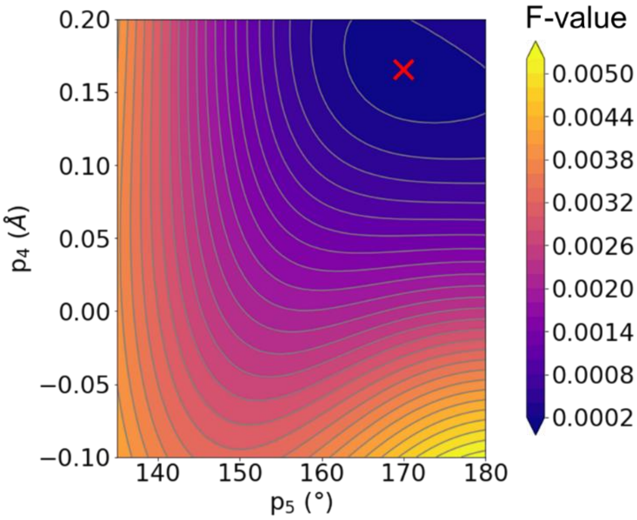

Figure S26: Fitting map (F-value) calculated for two parameters (p_4 and p_5) in model 3, maintaining the best-fit values for the remaining parameters fixed. The red cross indicates the best-fit value (F_{min}).

13 Reverse Monte Carlo simulations for the interpretation of Ni K-edge EXAFS data

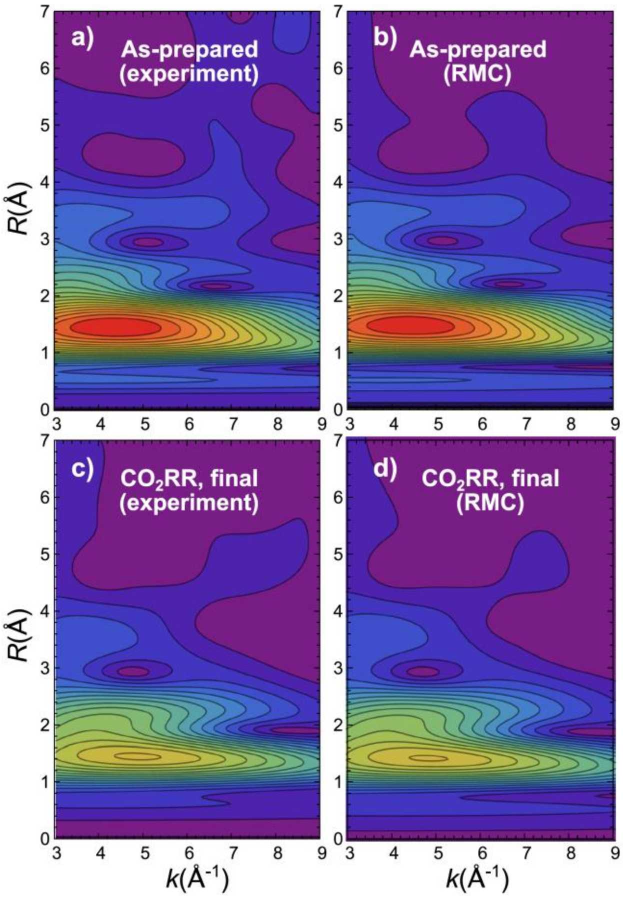

Figure S27: Results of the reverse Monte Carlo (RMC) simulations for the interpretation of Ni K-edge EXAFS spectra in HT-Ni-TMNC catalyst. Wavelet transforms of experimental (a, c) EXAFS spectra and of the theoretical EXAFS spectra calculated for the final structure model obtained in RMC simulations (b, d). Results for as-prepared (a, b) HT Ni-TMNC catalyst and for the final state of this catalyst under CO_2RR conditions (c, d) are shown.

14 XANES and EXAFS spectra of the Ni-TMNC catalyst initial and final states during CO_2RR

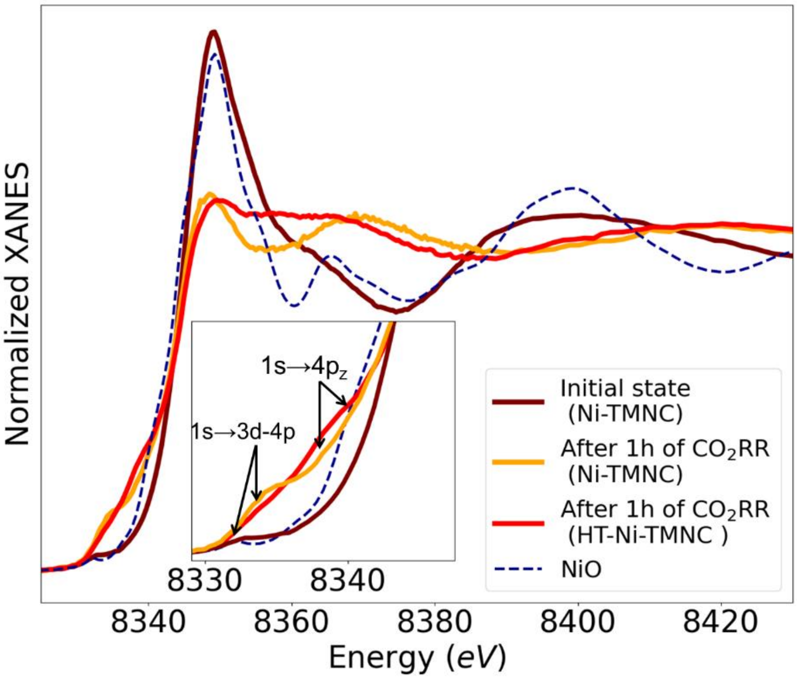

Figure S28: Ni K-edge experimental XANES spectra collected during operando CO_2RR conditions for the Ni-TMNC sample. The inset shows a magnification of the XANES pre-edge region. The measurements were performed in a CO_2-saturated 0.1 M $KHCO_3$ electrolyte under a static current density of -15.7 mA cm^{-2}.

15 Machine learning-assisted XANES fit of the initial and final states of Ni-TMNC sample

Despite the large difference in XANES features for HT Ni-TMNC and Ni-TMNC samples (**Figure S28**), we have found that for the both catalysts the final structure under CO_2RR conditions can be well described by model 3 (**Figure 6** in the main text).

On the other hand, interestingly, we found that model 1, which could successfully describe the as-prepared state of the HT Ni-TMNC catalyst, could not fit well the initial state of the Ni TMNC initial state (see **Figure S29** and **Table S11**), In particular, model 1 could not reproduce the very intense white line feature, characteristic for the as-prepared Ni-TMNC. We note that such a white line is characteristic for the octahedrally coordinated Ni(II)-O_6 species. It is plausible that, in presence of air, the Ni site in Ni-TMNC could be partially detached from the pyridines ring due to strong interaction with oxygen or adsorbed water species. Indeed, by removing the pyridines group from the structure model used to fit XANES spectrum for the as-prepared Ni-TMCN, the fit quality is improved.

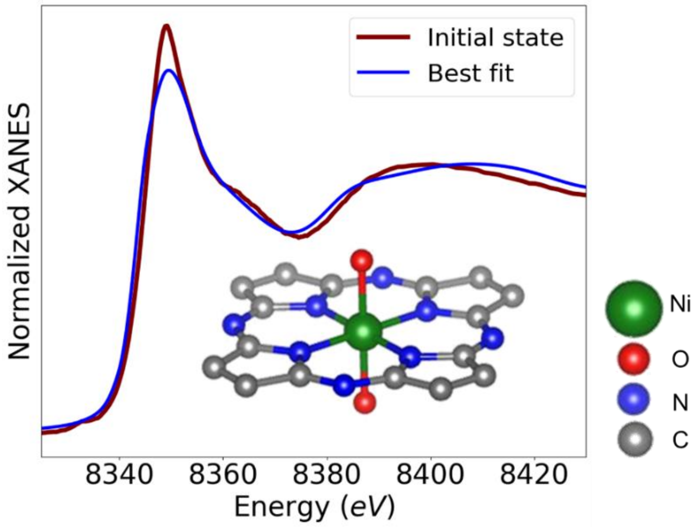

Figure S29: Comparison between the initial state XANES (beginning of the CO_2RR reaction) of the Ni-TMNC and its best-fit result obtained using Model 1 depicted in **Figure 6** of the main text. The inset shows the refined geometry. The refined structural parameters are reported in **Table S11**.

Table S11: Refined structural parameters obtained through the XANES fit shown in **Figure S29**.

Parameters	XANES Best Fit value
**Model 1 (Ni-TMNC initial state). Misfit (F_{min}):** 0.18 %	
p_1	0.12(3) Å
p_2	0.00(7) Å
P_3	-0.02(4) Å

Indeed, a simple octahedral structure model, where Ni is coordinated to six O atoms (**Figure S30**), provides a very good fit of the experimental XANES spectrum. Note that since the O and the N atoms are neighbours in the Periodic Table, we cannot distinguish between them reliably. Thus, while all six N neighbours in our model are represented by O atoms, in reality some bonding with N atoms cannot be excluded. The initial value of Ni-O distance in this model was 2.1 Å. The considered structure parameters that were optimized during the XANES fit of the data for the as-prepared Ni-TMNC sample are given in **Figure S30** and **Table S12**.

The results of XANES fitting, and the values of structure parameters obtained in XANES fits for Ni-TMNC sample are summarized in **Figure S31**, **Table S13** and **Table S14**.

Table S12: List of structural parameters shown in **Figure S30** employed for the fit of the Ni-TMNC initial state.

Parameter	Description	Range of variation
**Six coordinated Ni site (Precursor initial state)**		
p_1	Contraction/expansion of the in-plane Ni-O bonds.	[-0.2 : +0.2] Å
p_2	Contraction/expansions of the two axial Ni-O bonds.	[-0.2 : +0.2] Å

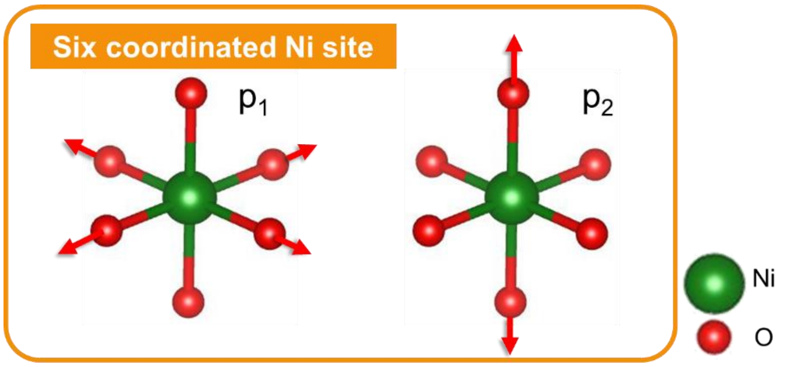

Figure S30: Set of possible structure model deformations considered in the fit of XANES spectra for the as-prepared Ni-TMNC sample.

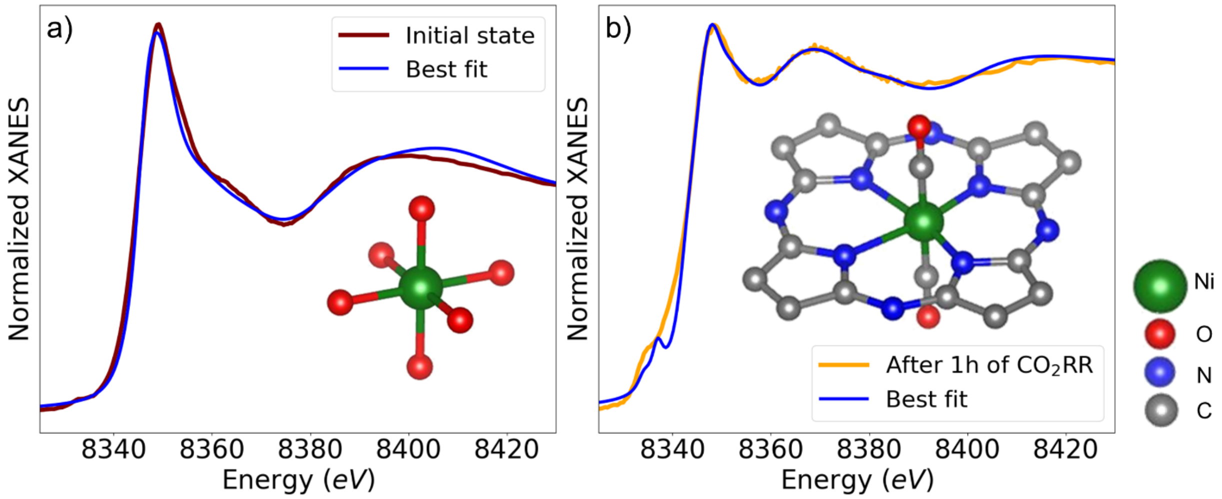

Figure S31: Comparison between the XANES Ni-TMNC spectra of the Ni-TMNC initial a) and final state after 1h of CO_2RR b) and their best-fit results. The insets display the refined geometries. The refined structural parameters are reported in **Table S13** and in **Table S14**.

For the as-prepared Ni-TMNC sample, the XANES best-fit results suggest that the axial Ni-O bonds are by ca. 0.1 Å shorter than the in-plane Ni-O bonds, suggesting a possible Jahn-Teller distortion. On the other hand, model 3 provides a good agreement with experimental data for the final spectrum of the working Ni-TMNC catalysts. We observe that all Ni-N and Ni-C/O distances for the final state of Ni-TMNC catalyst are similar to those in HT Ni-TMNC catalyst. The most significant differences are a larger expansion of the pyridines ring from the Ni site for the Ni-TMNC catalyst, which is almost by ca. 0.1 Å larger than that for HT Ni-TMNC catalyst, and more linear Ni-C-O chains ($\widehat{Ni - C - O}$ closer to 180 ° for the Ni-TMNC catalyst than for HT-Ni-TMNC).

Table S13: Refined structural parameters obtained through the XANES fits shown in **Figure S31**.

**Parameters**	**XANES Best Fit value**
**Six coordinated Ni site (Ni-TMNC initial state)**	
p_1	-0.11(2) Å
p_2	-0.2(1) Å
**Model 3 (Ni-TMNC final state, after 1h of CO_2RR)**	

p_1	0.17(1) Å
p_2	0.29(2) Å
p_3	-0.02(1) Å
p_4	0.17(2) Å
p_5	179.2(5) °

Table S14: Interatomic distances and Ni-CO ligand angle, calculated for the final structure models, obtained through the XANES fitting (**Figure S31**). The distances uncertainties derive from the ones showed in **Table S13**. For the details of the misfit quantity (F_{min}) calculations see **Section S7**.

Distances (average)/Angle	XANES Best Fit value
**Six coordinated Ni site (Ni-TMNC initial state). Misfit (F_{min}):** 0.09%	
Ni-O (planar O)	2.0(1) Å
Ni-O (vertical O)	1.92(2) Å
**Model 3 (Ni-TMNC final state, after 1h of CO_2RR). Misfit (F_{min}):** 0.02%	
Ni-C (C of the CO group)	1.73(1) Å
Ni-N (two pyridine N atoms closer to Ni)	1.89(2) Å
Ni-N (two pyridine N atoms more distant from Ni)	2.29(2) Å
C-O (C and O of the CO group)	1.27(2) Å
$Ni - \widehat{C} - O$ bond angle	179.2(5) °

16 References

(1) Timoshenko, J.; Roldan Cuenya, B. In Situ/Operando Electrocatalyst Characterization by X-ray Absorption Spectroscopy. *Chemical Reviews* **2021**, *121* (2), 882-961. DOI: 10.1021/acs.chemrev.0c00396.

(2) Biesinger, M. C.; Lau, L. W. M.; Gerson, A. R.; Smart, R. S. C. The role of the Auger parameter in XPS studies of nickel metal, halides and oxides. *Phys. Chem. Chem. Phys.* **2012**, *14* (7), 2434-2442, Article. DOI: 10.1039/c2cp22419d.

(3) Muralidharan, S.; Hayes, R. G. SATELLITES IN THE X-RAY PHOTOELECTRON-SPECTRA OF METALLOPORPHYRINS. *J. Chem. Phys.* **1979**, *71* (7), 2970-2974, Article. DOI: 10.1063/1.438700.

(4) Artyushkova, K. Misconceptions in interpretation of nitrogen chemistry from x-ray photoelectron spectra. *Journal of Vacuum Science & Technology A* **2020**, *38* (3), 1-8, Article. DOI: 10.1116/1.5135923.

(5) Artyushkova, K.; Kiefer, B.; Halevi, B.; Knop-Gericke, A.; Schlogl, R.; Atanassov, P. Density functional theory calculations of XPS binding energy shift for nitrogen-containing graphene-like structures. *Chemical Communications* **2013**, *49* (25), 2539-2541. DOI: 10.1039/c3cc40324f.

(6) Matanovic, I.; Artyushkova, K.; Strand, M. B.; Dzara, M. J.; Pylypenko, S.; Atanassov, P. Core Level Shifts of Hydrogenated Pyridinic and Pyrrolic Nitrogen in the Nitrogen-Containing Graphene-Based Electrocatalysts: In-Plane vs Edge Defects. *Journal of Physical Chemistry C* **2016**, *120* (51), 29225-29232. DOI: 10.1021/acs.jpcc.6b09778.

(7) Ortega, K. F.; Arrigo, R.; Frank, B.; Schlogl, R.; Trunschke, A. Acid-Base Properties of N-Doped Carbon Nanotubes: A Combined Temperature-Programmed Desorption, X-ray Photoelectron Spectroscopy, and 2-Propanol Reaction Investigation. *Chemistry of Materials* **2016**, *28* (19), 6826-6839, Article. DOI: 10.1021/acs.chemmater.6b01594.

(8) Ott, S.; Du, F.; Luna, M. L.; Dao, T. A.; Selve, S.; Roldan Cuenya, B.; Orfanidi, A.; Strasser, P. Property-reactivity relations of N-doped PEM fuel cell cathode catalyst supports. *Appl. Catal. B-Environ.* **2022**, *306*, 1-11. DOI: 10.1016/j.apcatb.2022.121118.

(9) Spath, F.; Zhao, W.; Gleichweit, C.; Gotterbarm, K.; Bauer, U.; Hofert, O.; Steinruck, H. P.; Papp, C. Hydrogenation and dehydrogenation of nitrogen-doped graphene investigated by X-ray photoelectron spectroscopy. *Surf. Sci.* **2015**, *634*, 89-94, Article. DOI: 10.1016/j.susc.2014.11.009.

(10) Timoshenko, J.; Kuzmin, A. Wavelet data analysis of EXAFS spectra. *Computer Physics Communications* **2009**, *180* (6), 920-925, Article. DOI: 10.1016/j.cpc.2008.12.020.

(11) Funke, H.; Chukalina, M.; Scheinost, A. C. A new FEFF-based wavelet for EXAFS data analysis. *Journal of Synchrotron Radiation* **2007**, *14*, 426-432, Article. DOI: 10.1107/s0909049507031901.
(12) Martini, A.; Signorile, M.; Negri, C.; Kvande, K.; Lomachenko, K. A.; Svelle, S.; Beato, P.; Berlier, G.; Borfecchia, E.; Bordiga, S. EXAFS wavelet transform analysis of Cu-MOR zeolites for the direct methane to methanol conversion. *Phys. Chem. Chem. Phys.* **2020**, *22* (34), 18950-18963, Article. DOI: 10.1039/d0cp01257b.
(13) Ankudinov, A. L.; Ravel, B.; Rehr, J. J.; Conradson, S. D. Real-space multiple-scattering calculation and interpretation of x-ray-absorption near-edge structure. *Phys. Rev. B* **1998**, *58* (12), 7565-7576, Article. DOI: 10.1103/PhysRevB.58.7565.
(14) Beauchemin, S.; Hesterberg, D.; Beauchemin, M. Principal component analysis approach for modeling sulfur K-XANES spectra of humic acids. *Soil Science Society of America Journal* **2002**, *66* (1), 83-91, Article.
(15) Martini, A.; Guda, A. A.; Guda, S. A.; Bugaev, A. L.; Safonova, O. V.; Soldatov, A. V. Machine learning powered by principal component descriptors as the key for sorted structural fit of XANES. *Phys. Chem. Chem. Phys.* **2021**, *23* (33), 17873-17887, Article. DOI: 10.1039/d1cp01794b.
(16) Wang, Q.; Hanson, J. C.; Frenkel, A. I. Solving the structure of reaction intermediates by time-resolved synchrotron x-ray absorption spectroscopy. *J. Chem. Phys.* **2008**, *129* (23), 7, Article. DOI: 10.1063/1.3040271.
(17) Guda, S. A.; Guda, A. A.; Soldatov, M. A.; Lomachenko, K. A.; Bugaev, A. L.; Lamberti, C.; Gawelda, W.; Bressler, C.; Smolentsev, G.; Soldatov, A. V.; et al. Optimized Finite Difference Method for the Full-Potential XANES Simulations: Application to Molecular Adsorption Geometries in MOFs and Metal-Ligand Intersystem Crossing Transients. *Journal of Chemical Theory and Computation* **2015**, *11* (9), 4512-4521, Article. DOI: 10.1021/acs.jctc.5b00327.
(18) Hedin, L.; Lundqvist, S. Effects of Electron-Electron and Electron-Phonon Interactions on the One-Electron States of Solids. In *Solid State Physics*, Seitz, F., Turnbull, D., Ehrenreich, H. Eds.; Vol. 23; Academic Press, 1970; pp 1-181.
(19) von Barth, U.; Hedin, L. A local exchange-correlation potential for the spin polarized case. i. *Journal of Physics Part C Solid State Physics* **1972**, *5* (13), 1629-+, Article. DOI: 10.1088/0022-3719/5/13/012.
(20) Joly, Y. FDMNES manual. *http://fdmnes.neel.cnrs.fr/* **2021**, *FDMNES manual*, Manual.
(21) Avakyan, L. A.; Manukyan, A. S.; Mirzakhanyan, A. A.; Sharoyan, E. G.; Zubavichus, Y. V.; Trigub, A. L.; Kolpacheva, N. A.; Bugaev, L. A. Atomic Structure of Nickel Phthalocyanine Probed by X-Ray Absorption Spectroscopy and Density Functional Simulations. *Optics and Spectroscopy* **2013**, *114* (3), 347-352, Article. DOI: 10.1134/s0030400x1303003x.
(22) Wright, S. J. Coordinate descent algorithms. *Mathematical Programming* **2015**, *151* (1), 3-34, Article; Proceedings Paper. DOI: 10.1007/s10107-015-0892-3.
(23) Smolentsev, G.; Soldatov, A. V.; Feiters, M. C. Three-dimensional local structure refinement using a full-potential XANES analysis. *Phys. Rev. B* **2007**, *75* (14), 5, Article. DOI: 10.1103/PhysRevB.75.144106.
(24) Guda, A. A.; Guda, S. A.; Lomachenko, K. A.; Soldatov, M. A.; Pankin, I. A.; Soldatov, A. V.; Braglia, L.; Bugaev, A. L.; Martini, A.; Signorile, M.; et al. Quantitative structural determination of active sites from in situ and operando XANES spectra: From standard ab initio simulations to chemometric and machine learning approaches. *Catalysis Today* **2019**, *336*, 3-21, Article; Proceedings Paper. DOI: 10.1016/j.cattod.2018.10.071.
(25) Kozyr, E. G.; Bugaev, A. L.; Guda, S. A.; Guda, A. A.; Lomachenko, K. A.; Janssens, K.; Smolders, S.; De Vos, D.; Soldatov, A. V. Speciation of Ru Molecular Complexes in a Homogeneous Catalytic System: Fingerprint XANES Analysis Guided by Machine Learning. *Journal of Physical Chemistry C* **2021**, *125* (50), 27844-27852, Article. DOI: 10.1021/acs.jpcc.1c09082.
